# RUNX2 is essential for maintaining synchondrosis chondrocytes and cranial base growth

**DOI:** 10.1038/s41413-025-00426-z

**Published:** 2025-05-29

**Authors:** Shawn A. Hallett, Ashley Dixon, Isabella Marrale, Lena Batoon, José Brenes, Annabelle Zhou, Ariel Arbiv, Vesa Kaartinen, Benjamin Allen, Wanida Ono, Renny T. Franceschi, Noriaki Ono

**Affiliations:** 1 Department of Periodontics and Oral Medicine, University of Michigan School of Dentistry, Ann Arbor, MI USA; 2 Department of Biological and Materials Sciences and Prosthodontics, University of Michigan School of Dentistry, Ann Arbor, MI USA; 3 Department of Cell and Developmental Biology, University of Michigan Medical School, Ann Arbor, MI USA; 4 Department of Orthodontics, University of Texas Health Science Center at Houston School of Dentistry, Houston, TX USA; 5 Department of Diagnostic and Biomedical Sciences, University of Texas Health Science Center at Houston School of Dentistry, Houston, TX USA

**Keywords:** Bone, Homeostasis

## Abstract

The cranial base synchondroses, comprised of opposite-facing bidirectional chondrocyte layers, drive anteroposterior cranial base growth. In humans, *RUNX2* haploinsufficiency causes cleidocranial dysplasia associated with deficient midfacial growth. However, how RUNX2 regulates chondrocytes in the cranial base synchondroses remains unknown. To address this, we inactivated *Runx2* in postnatal synchondrosis chondrocytes using a tamoxifen-inducible *Fgfr3-creER* (Fgfr3-Runx2^cKO^) mouse model. Fgfr3-Runx2^cKO^ mice displayed skeletal dwarfism and reduced anteroposterior cranial base growth associated with premature synchondrosis ossification due to impaired chondrocyte proliferation, accelerated hypertrophy, apoptosis, and osteoclast-mediated cartilage resorption. Lineage tracing reveals that *Runx2*-deficient Fgfr3^+^ cells failed to differentiate into osteoblasts. Notably, *Runx2*-deficient chondrocytes showed an elevated level of FGFR3 and its downstream signaling components, pERK1/2 and SOX9, suggesting that RUNX2 downregulates FGFR3 in the synchondrosis. This study unveils a new role of *Runx2* in cranial base chondrocytes, identifying a possible RUNX2-FGFR3-MAPK-SOX9 signaling axis that may control cranial base growth.

## Introduction

The craniofacial complex comprises the neurocranium and viscerocranium, which encase the central nervous system and the upper airway, respectively. The neurocranium is composed of the cranial base and the cranial vault. The cranial base comprises segmented bones, including the ethmoid, pre-sphenoid, basisphenoid, basioccipital, and exoccipital.^1^ The anterior cranial base is derived from the neural crest while the posterior aspect is derived from the paraxial mesoderm.^2^ The cranial base bones are separated by the synchondroses, cartilaginous growth centers comprised of chondrocytes that undergo conversion into osteoblasts and drive cranial base lengthening. The inter-sphenoid synchondrosis (ISS) is located between the pre-sphenoid and basisphenoid bones, the more posterior spheno-occipital synchondrosis (SOS) lies between the basisphenoid and basioccipital bones, and the anterior/posterior intra-occipital (AIOS/PIOS) synchondroses lie between the basioccipital, exoccipital and occipital bones. The SOS and ISS function to promote the growth of the cranial base while the AIOS/PIOS direct the growth of the foramen magnum. Explant studies demonstrate that the SOS maintains its innate chondrogenesis capacity even in the absence of external functional activities such as the growth of the central nervous system or the oropharyngeal-nasopharyngeal space.^3–5^ Thus, the synchondroses have been postulated to function as growth centers of the craniofacial complex. The SOS is the largest and contributes most significantly to cranial base growth. In humans, the ISS ossifies at 2–3 years of age, while the PIOS and AIOS ossify at 4–6 and 6–8 years, respectively; in contrast, the SOS ossifies much later, between 16–18 years.^6–8^ In humans, ~60 percent of cranial base growth occurs embryonically, and 40 percent occurs postnatally, extending into adolescence.^9^ This underscores the significance of the SOS as a potential target for pharmacological intervention in conditions associated with premature ossification of the synchondroses. In mice, the ISS completely ossifies between postnatal day 180 (P180) to P390, while the SOS fuses by P390. Notably, the ISS and SOS exhibit initial mineralization in the central portion of the cartilage at P60 and P21, respectively. In contrast, the PIOS ossifies by P30.^10^ Moreover, the premature fusion of the PIOS and AIOS has been linked to reduced foramen magnum size, including foramen magnum stenosis in syndromic craniosynostoses and achondroplasia.^11,12^ Like the long bone growth plate, synchondroses consist of distinct layers of round, proliferating, and hypertrophic chondrocytes. However, unlike the unidirectional organization of chondrocytes observed in the growth plate,^13,14^ each synchondrosis contains two growth plate-like structures with a mirror image orientation. Despite the critical role the synchondrosis plays in cranial base growth, the mechanisms governing its molecular regulation remain largely unknown.^15–17^

Deficient cranial base growth in humans is associated with reduced midfacial growth,^18^ skeletal Class III malocclusion^19^ and foramen stenosis,^12^ most prominently found in syndromic craniofacial deformities, including Apert, Pfeiffer, and Crouzon^20,21^ as well as Down and Klinefelter syndromes.^22,23^ Additionally, genetic disorders affecting the skeleton often cause cranial base malformations. For instance, achondroplasia, caused by activating mutations in *FGFR3*, results in premature fusion of the long bone growth plate and cranial base synchondroses, and nasal anteversion, leading to dwarfism and deficient cranial base growth, respectively.^24^ These conditions often pose challenges to breathing, speaking, and chewing. Surgical interventions such as Le Fort osteotomy are the primary treatments for correcting these structural skeletal deficiencies.^25^ Understanding the molecular mechanisms regulating cranial base growth is crucial for improved treatments of skeletal Class III malocclusion as well as foramen magnum stenosis.

Individuals with non-syndromic skeletal Class III malocclusion can also exhibit midfacial retrusion associated with the truncation of the cranial base.^26^ It is postulated that the collective activities of cranial base chondrocytes determine the rate of anteroposterior elongation of the midface by promoting cranial base growth. However, the molecular mechanisms regulating these processes remain largely unknown.

RUNX2, a master regulator of osteoblast differentiation, plays a crucial role in craniofacial development.^27,28^ *RUNX2* haploinsufficiency in humans leads to cleidocranial dysplasia (CCD), characterized by hypoplastic clavicles, patent sutures and fontanelles, supernumerary teeth, and short stature.^29,30^ CCD patients often exhibit deficient cranial base growth, potentially attributed to reduced cartilage growth in the synchondroses.^31^ RUNX2 is expressed in cranial base synchondrosis chondrocytes and the anterior palate.^27^ *Runx2* global heterozygous loss-of-function mutant mice show a dome-shaped skull and abnormal synchondroses,^32^ suggesting RUNX2 may also play critical roles in cranial base development. However, how *Runx2* functions in cranial base synchondrosis chondrocytes remains unknown.

The *FGFR3*^*G380R*^ activating point mutation, associated with the most common form of human dwarfism, achondroplasia, leads to the shortening of both long and craniofacial bones due to premature growth plate hypertrophy and synchondrosis fusion.^33,34^ Similarly, constitutive activation of the downstream FGFR3 effector, MEK1, in chondrocytes^35,36^ causes premature synchondrosis fusion, mirroring the phenotype observed in *Fgfr3*^*G374R*^ mice, which models the orthologous human mutation. Consequently, FGFR3-MAPK signaling promotes synchondrosis growth and fusion, leading to similar craniofacial phenotypes observed with *RUNX2* mutations in CCD. Interestingly, in vitro studies show that RUNX2 can directly regulate *Fgfr3* transcription and downstream MAPK signaling,^37^ suggesting a potential link between RUNX2 actions and FGFR3 activities. However, how RUNX2 regulates FGFR3 in vivo has yet to be determined.

Here, we utilize in vivo lineage-tracing and conditional deletion approaches to define the requirement of *Runx2* in cranial base growth. As will be shown, RUNX2 functions in *Fgfr3*-positive synchondrosis chondrocytes and regulates cranial base growth. Fgfr3-Runx2^cKO^ synchondrosis chondrocytes lack proliferative capabilities and prematurely undergo apoptosis and hypertrophy. This is associated with increased *Rankl* levels stimulating osteoclastogenesis and subsequent synchondrosis degradation followed by premature ossification. In addition, we also found that RUNX2 is a possible negative regulator of FGFR3, a finding that may relate RUNX2 deficiency to abnormally enhanced FGFR3 activities. These findings highlight the complementary roles of RUNX2 and FGFR3 signaling in cranial base growth, which may provide novel therapeutic targets to treat cranial base skeletal defects.

## Results

### *Fgfr3-creER* labels chondrocytes and their precursors in postnatal cranial base synchondroses

**Fig. 1:**
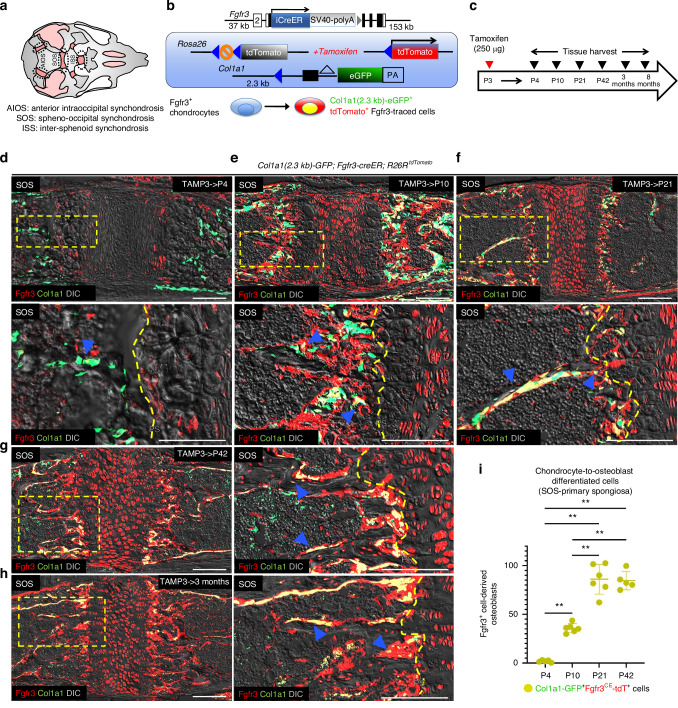
*Fgfr3-creER* labels chondrocytes and their precursors in postnatal cranial base synchondrosis. **a** Cartoon illustration of cranial base spheno-occipital synchondrosis (SOS), inter-sphenoid synchondrosis (ISS), and anterior intra-occipital synchondrosis (AIOS) housed within the craniofacial complex. Pink: cartilage, gray: bone. **b** *Fgfr3-creER*; *R26R*-*tdTomato* lineage-tracing model combined with *Col1a1(2.3* *kb)-eGFP* reporter. Single intraperitoneal injection of tamoxifen at P3 induces *cre* recombination, leading to the labeling of Fgfr3^+^ cells by tdTomato. If these cells differentiate into osteoblastic cells, they become simultaneously marked by Col1a1(2.3 kb)-eGFP. **c** Timeline for the lineage-tracing experiments. Tamoxifen injection (250 μg) at P3 and lineage-traced to P4, P10, P21, P42, 3 months and 8 months. Lineage-tracing of Fgfr3^CE^-tdT^+^ SOS cells at P4 (**d**) P10 (**e**) P21 (**f**) P42 (**g**) and 3 months (**h**). The boxed region is shown in higher magnification. Blue arrowheads represent Col1a1(2.3 kb)-GFP^+^ osteoblasts derived from Fgfr3^CE^-tdT^+^ chondrocytes at primary spongiosa. Red: Fgfr3^CE^-tdT, green: Col1a1(2.3 kb)-GFP, yellow: Col1a1(2.3 kb)-GFP^+^Fgfr3^CE^-tdT^+^, gray: DIC. PS: primary spongiosa. Scale bar: 100 µm. **i** Quantification of Col1a1(2.3 kb)-GFP^+^Fgfr3^CE^-tdT^+^ cells at primary spongiosa at P4 (*n* = 5), P10 (*n* = 6), P21 (*n* = 6) and P42 (*n* = 5) collected from serial sections of the SOS. **P < 0.01, Mann-Whitney’s *U*-test. Data are presented as mean ± s.d

Chondrocytes in the synchondroses are the primary driver of cranial base growth (Fig. 1a). To inactivate *Runx2* in postnatal cranial base chondrocytes, we utilized a tamoxifen-inducible *Fgfr3-creER* P1-derived artificial chromosome (PAC) transgenic line.^38^ We recently showed that *Fgfr3-creER* is active in long bones in chondrocytes and periosteal and endosteal stromal cells.^39^ First, to define the cell population targeted by *Fgfr3-creER* in the cranial base synchondroses, we crossed *Fgfr3-creER* with *R26R*^*tdTomato*^ and *Col1a1(2.3* *kb)-GFP* reporter lines to visualize the cell lineage progression of Fgfr3^+^ cells toward osteoblasts during postnatal cranial base growth (Fig. 1b). *Col1a1(2.3* *kb)-GFP*; *Fgfr3-creER; R26R*^*tdTomato*^ mice received a single dose of tamoxifen at postnatal day 3 (P3) to transiently activate *cre-lox* recombination and permanently label Fgfr3^+^ cells with tdTomato (Fgfr3^CE^-tdT^+^ cells). The injected mice were lineage-traced for various periods and collected at P4, P10, P21, and P42, postnatal months 3 and 8 (3/8 months) (Fig. 1c).

Following one day of lineage-tracing at P4 (short-trace), Fgfr3^CE^-tdT^+^ cells were distributed across layers of the SOS and ISS in low numbers (Fig. 1d, upper/lower panels; Figs. S1a and S2a, g); these cells were distinct from osteoblasts, with very few Col1a1-GFP^+^Fgfr3^CE^-tdT^+^ cells at the primary spongiosa (Fig. 1d, lower panel, arrowhead; Fig. 1i). By P10, chondrocytes of all layers of the synchondroses were marked as Fgfr3^CE^-tdT^+^ (Fig. 1e, upper panel; Fig. S2b, left panel), which began to differentiate into osteoblasts at the primary spongiosa (Fig. 1e, lower panel, arrowheads and Fig. S2b, right panel, arrowhead; quantification in Figs. 1i and S2f), a behavior consistent with that observed in the postnatal long bone growth plate.^40^ This trend continued to P21 in both the SOS, ISS, and AIOS (Fig. S3a). By this time, Col1a1-GFP^+^Fgfr3^CE^-tdT^+^ osteoblasts were abundant in the primary spongiosa and cortical bones (Figs. 1f and S2c, arrowheads; Fig. S3a, right panel, arrowheads). At P42 and continuing onto 3 months, Col1a1-GFP^+^Fgfr3^CE^-tdT^+^ osteoblasts remained within the spongiosa (Figs. 1g, h and S2d, e). Following lineage-tracing to 8 months, Fgfr3^CE^-tdT^+^ cells continued to be present as proliferating, pre-hypertrophic, and hypertrophic chondrocytes, as well as cortical osteoblasts, suggesting that Fgfr3^+^ cells continue to contribute to cranial base synchondrosis chondrocytes in adulthood (Fig. S1c). However, while the SOS remains patent at 8 months, the chondrocyte layers of the SOS were shortened, disorganized, and associated with possible mineralization within the central portion (Fig. S1c, arrowheads). Quantification of Col1a1-GFP^+^Fgfr3^CE^-tdT^+^ cells in the primary spongiosa showed that Fgfr3^+^ cell-derived osteoblasts gradually increased from P4 to P21 but remained consistent from P21 to P42 in the areas adjoining the SOS and ISS (Figs. 1i and S2f). In contrast, Fgfr3^CE^-tdT^+^ chondrocytes generally increased from P4 to P42 in both synchondroses (Figs. S1a and S2g). Moreover, Col1a1-GFP^+^ single-labeled chondrocytes increased from P4 to P42 in the SOS but remained constant in the ISS at all time points (Figs. S1b and S2h), presumably reflecting the late timing that the SOS matures and ossifies.

Thus, *Fgfr3-creER* robustly labels chondrocytes and their precursors in the cranial base synchondroses during postnatal cranial base growth, which readily differentiate into osteoblasts. Furthermore, Fgfr3^CE^-tdT^+^ cells persist within the synchondrosis and its surrounding bony regions for an extended period during adult stages.

### *Runx2* inactivation in Fgfr3^+^ cells causes craniofacial skeletal abnormalities

**Fig. 2:**
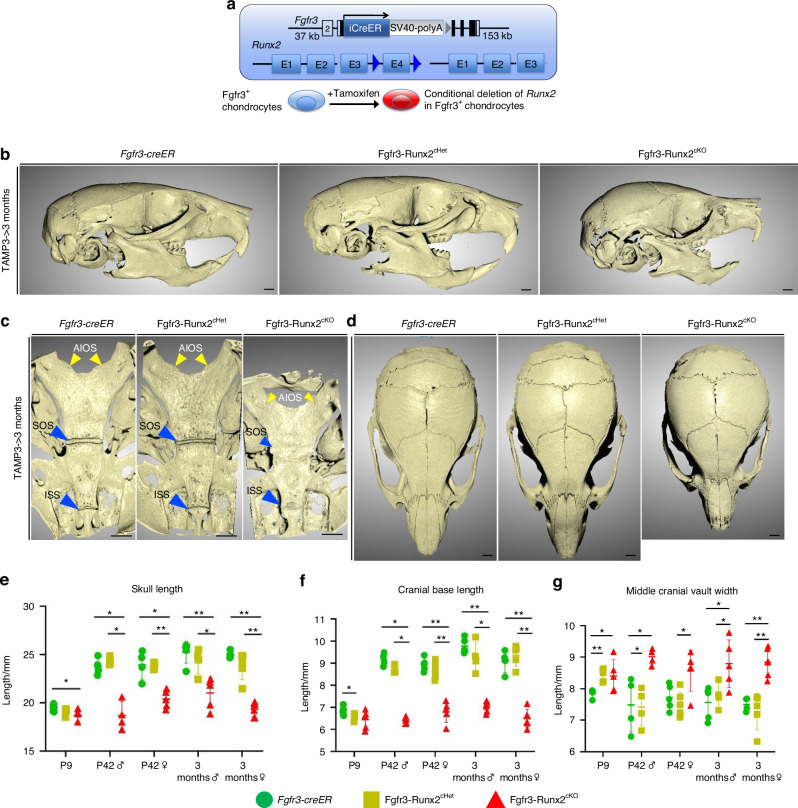
*Runx2* inactivation in Fgfr3^+^ cells causes craniofacial skeletal abnormalities. **a** *Fgfr3-creER*; *R26R*-*tdTomato* mice were crossed with mice carrying a *runt-related transcription factor 2* (*Runx2*) floxed allele (flanking exon 4). Single intraperitoneal injection of tamoxifen at P3 induces *cre* recombination, leading to heterozygous or homozygous knockout of *Runx2* in Fgfr3^+^ chondrocytes [*Fgfr3-creER; Runx2*^*fl/+*^ (Fgfr3-Runx2^cHet^)/*Fgfr3-creER; Runx2*^*fl/fl*^ (Fgfr3-Runx2^cKO^)]. 3D renderings of Control (*Fgfr3-creER*), Fgfr3-Runx2^cHet^, and Fgfr3-Runx2^cKO^ mice at 3 months indicate decreased anteroposterior growth of entire craniofacial complex (**b**), premature ossification of the synchondroses (**c**), and widened cranial vaults (**d**) in Fgfr3-Runx2^cKO^ animals. Quantification of skull length (**e**), cranial base length (**f**), and middle cranial vault width (**g**) at P9 [Control (*n* = 6)], Fgfr3-Runx2^cHet^ (*n* = 5), Fgfr3-Runx2^cKO^ (*n* = 5), P42 [male-Control (*n* = 4), Fgfr3-Runx2^cHet^ (*n* = 4), Fgfr3-Runx2^cKO^ (*n* = 4); female-Control (*n* = 5), Fgfr3-Runx2^cHet^ (*n* = 6), Fgfr3-Runx2^cKO^ (*n* = 5)] and 3 months [male-Control (*n* = 5), Fgfr3-Runx2^cHet^ (*n* = 4), Fgfr3-Runx2^cKO^ (*n* = 5); female-Control (*n* = 4), Fgfr3-Runx2^cHet^ (*n* = 5), Fgfr3-Runx2^cKO^ (*n* = 6)]. *P < 0.05, **P < 0.01, Mann-Whitney’s *U*-test. Data are presented as mean ± s.d. Scale bar = 1 mm

Subsequently, we utilized a conditional deletion approach to define *Runx2* function in cranial base synchondrosis chondrocytes. For this purpose, we generated littermates of control (*Fgfr3-creER; Runx2*^*+/+*^), *Runx2* conditional heterozygous [*Fgfr3-creER; Runx2*^*fl/+*^ (Fgfr3-Runx2^cHet^)] and homozygous mutant [*Fgfr3-creER; Runx2*^*fl/fl*^ (Fgfr3-Runx2^cKO^)] mice, which were injected by a single dose of tamoxifen at P3 (Fig. 2a). Fgfr3-Runx2^cKO^ mice displayed skeletal dwarfism associated with reduced body weight, and naso-anal and tail lengths at P42 and 3 months in both sexes (Fig. S4a, b–d). To determine the overall impact of *Runx2* inactivation in Fgfr3^+^ cells on cranial base growth, we performed linear measurements on three-dimensional micro-CT (3D-μCT) scans of P9, P42, and 3 months-old male and female skulls using previously described landmarks (Fig. S4e).^41^ Notably, Fgfr3-Runx2^cKO^ skulls failed to elongate along the anteroposterior (AP) axis in both sexes between P9 and P42, during which the Control skulls substantially elongated (~20%) (Fig. 2b, left panel, e). These changes were associated with decreased cranial vault and facial lengths in Fgfr3-Runx2^cKO^ mice (Fig. S4f, g). Further, we evaluated craniofacial proportions by assessing the ratios of the lengths, heights, and widths of individual skeletal elements in anterior (facial) and posterior (cranial vault) regions. We observed slight increases in the ratio of facial length to facial height in P42 Fgfr3-Runx2^cKO^ female skulls (Fig. S4h). The ratios of anterior cranial vault width to facial height were increased, and facial length to anterior cranial vault width was decreased in Fgfr3-Runx2^cKO^ skulls at all time points and sexes (Fig. S4i, j). The ratios of cranial vault length to height were decreased in Fgfr3-Runx2^cKO^ P42 males and both sexes at 3 months (Fig. S4k), while middle cranial vault widths to heights were increased at P42 and 3 months in both sexes (Fig. S4l). We observed decreases in the cranial vault length to middle cranial vault width at all time points and in both sexes in Fgfr3-Runx2^cKO^ animals (Fig. S4m). Moreover, 3 months Fgfr3-Runx2^cKO^ skulls also displayed defective ossification of the frontal and zygomatic bones and mandibular hypoplasia associated with reduced incisor length (Fig. 2b, right panel). These data were confirmed morphologically using 3D-μCT analyses (Fig. S5a, b) and demonstrate that craniofacial skeletal proportions were significantly altered in Fgfr3-Runx2^cKO^ mice. We observed mild premature fusion of the coronal suture intermittently in several Fgfr3-Runx2^cKO^ P42 males (Fig. S5b, right panel, arrowhead). Interestingly, Fgfr3-Runx2^cHet^ skulls intermittently displayed mild differences in cranial vault shape at P9 that were no longer apparent by P42 in both sexes (Fig. S5a, middle panels).

We further closely examined the synchondroses by 3D-μCT analyses. The Fgfr3-Runx2^cKO^ SOS was patent at P9 (Fig. S6a, upper/lower right panels, maize arrowheads) but became prematurely ossified at P42 (Fig. S6b, upper/lower right panels, maize arrowheads) and 3 months (Fig. 2c, right panel, blue arrowheads). In contrast, the Fgfr3-Runx2^cKO^ ISS was patent at P9 (Fig. S6a, upper/lower right panels, blue arrowheads) but mineralized at P42 in a manner depressed relative to the surfaces of the pre-sphenoid and basisphenoid bones (Fig. S6b, upper/lower right panels, blue arrowheads) and 3 months (Fig. 2c, right panel, arrowheads). At 3 months, we observed mild decreases in SOS and ISS lengths in Fgfr3-Runx2^cKO^ synchondroses in males, with converse increases in ISS lengths in females (Fig. S6f, g). These changes resulted in decreases in whole cranial base length, including lengths of individual cranial base bones (i.e., basioccipital, basisphenoid, and pre-sphenoid) at P42 and 3 months (Figs. 2f and S6c–e). Three-dimensional μCT analysis revealed minimal morphological changes of the AIOS, foramen magnum, and exoccipital bone of Fgfr3-Runx2^cKO^ mice at all time points (Fig. S7a, arrowheads, b, right panels). The AIOS was completely fused by P42 in both Fgfr3-Runx2^cHet^ and Fgfr3-Runx2^cKO^ mice (Fig. S7a, middle panels, arrowheads). Similarly, linear measurements of the AIOS and foramen magnum widths and exoccipital length (Fig. S7c) revealed moderate differences (Fig. S7d–e). Thus, Fgfr3-Runx2^cKO^ mice exhibit minimal phenotypes in the posterior skull, including the AIOS, exoccipital bone, and foramen magnum.

Interestingly, the lengths of the middle cranial vault were increased in Fgfr3-Runx2^cKO^ mice at all time points, while the lengths of the anterior cranial vault increased at P42 and 3 months, resulting in a dome-shaped skull typically seen in syndromic craniosynostoses (Fig. 2d, right panel, g; Fig. S5b, right panels; Fig. S8a middle/right panels, c).

These results demonstrate that *Runx2* inactivation in Fgfr3^+^ synchondrosis chondrocytes causes an arrest in anteroposterior growth of the skull associated with premature synchondrosis ossification. Given the minor differences observed between control and Fgfr3-Runx2^cHet^ craniofacial parameters, the remainder of our studies focuses on the comparison of Fgfr3-Runx2^cHet^ (Control) and Fgfr3-Runx2^cKO^ synchondroses in both sexes.

### *Runx2* inactivation in Fgfr3^+^ cells disrupts cell organization of the cranial base synchondroses

**Fig. 3:**
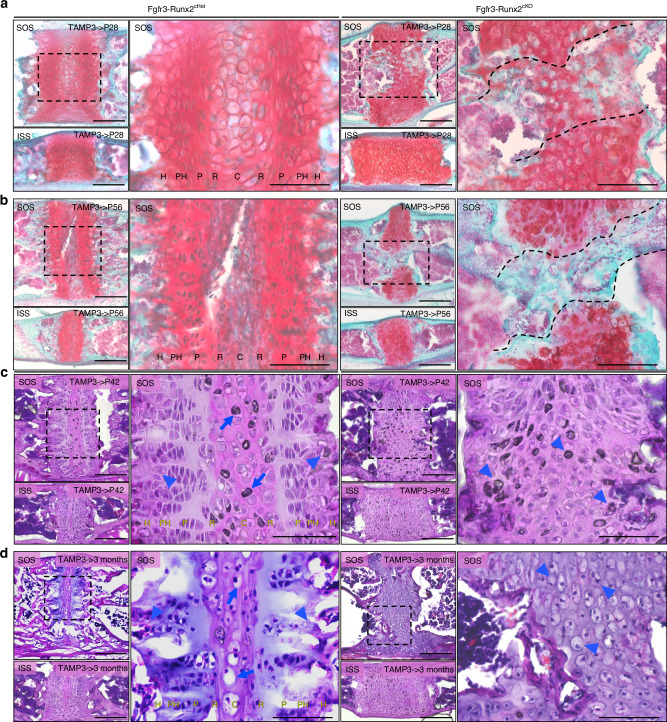
*Runx2* inactivation in Fgfr3^+^ cells disrupts cell organization of the synchondrosis. Fgfr3-Runx2^cHet^ and Fgfr3-Runx2^cKO^ mice were injected with a single dose of tamoxifen once at P3 and euthanized at P28 (**a**) and P56 (**b**). Safranin-O staining revealed organized chondrocyte layers in Fgfr3-Runx2^cHet^ mice. Boxed regions show higher magnification. Fgfr3-Runx2^cKO^ mice displayed a progressive fusion of the central portion of the SOS (**a**, **b** right magnified panels) while the ISS was widened. Black dashed lines highlight the fusion defect. Fgfr3-Runx2^cHet^ and Fgfr3-Runx2^cKO^ mice were injected with tamoxifen once at P3 and euthanized at P42 (**c**) and 3 months (**d**). Hematoxylin and eosin staining revealed organized chondrocyte layers in Fgfr3-Runx2^cHet^ mice, showing higher magnification in boxed regions. Fgfr3-Runx2^cKO^ mice displayed bone formation areas throughout the SOS’s central portion (**c**, **d** right panels). Arrows and arrowheads indicate hypertrophic-like cells in the central and hypertrophic zones of SOS, respectively (**c**, **d** left magnified panels) and areas of chondrocyte disorganization in the Fgfr3-Runx2^cKO^ SOS (**c**, **d** right magnified panels). C central zone, R resting zone, P proliferating zone, PH pre-hypertrophic zone, H hypertrophic zone. Scale bar = 100 µm

Next, we performed histological analyses to define the basis for the observed premature ossification of the synchondroses. Fgfr3-Runx2^cHet^ and Fgfr3-Runx2^cKO^ mice were analyzed at the above-described time points. Safranin-O staining was performed on sections of the SOS and ISS at P28 and P56 to visualize the progressive organization of the synchondroses during postnatal development (Fig. 3a, b, left magnified panels). In Fgfr3-Runx2^cHet^ mice, cartilage formation was bilaterally distributed into resting, proliferating, pre-hypertrophic, and hypertrophic zones (Fig. 3a, b, left magnified panels). Hematoxylin and eosin (H&E) staining similarly revealed that Fgfr3-Runx2^cHet^ mice analyzed at P42 and 3 months displayed synchondroses with well-organized bidirectional layers of resting, proliferating, pre-hypertrophic and hypertrophic chondrocytes (Fig. 3a–d, left panels). In contrast, Fgfr3-Runx2^cKO^ synchondroses displayed a derangement of tissue architecture, wherein abnormally shaped bulbous cells with hypertrophic chondrocyte-like morphology were predominant (Fig. 3c, d, right panels, arrowheads). This was associated with progressive degradation of the central portion of the SOS, denoted by the formation of a “bony bridge” accompanied by premature fusion of the SOS (Fig. 3a, b, right magnified panels, black lines). The ISS was also widened and populated by hypertrophic chondrocyte-like cells in Fgfr3-Runx2^cKO^ mice (Fig. 3a, b, right lower panels). Interestingly, the AIOS also exhibited disorganization of chondrocyte layers at P21 in Fgfr3-Runx2^cKO^ mice (Fig. S9a, right magnified panel, arrowheads).

To define the recombination efficiency of the *Runx2*^*fl/fl*^ allele, we assessed phosphorylated RUNX2 levels [P-S319-Runx2 (pRUNX2)] in Fgfr3-Runx2^cHet^ and Fgfr3-Runx2^cKO^ synchondroses using immunohistochemistry. This antibody recognizes the activated form of RUNX2.^42^ pRUNX2^+^Fgfr3^CE^-Control^+^ chondrocytes were present in the proliferating zone of Fgfr3-Runx2^cHet^ synchondroses but were nearly absent in Fgfr3-Runx2^cKO^ synchondroses, demonstrating the validity of *Runx2* inactivation in Fgfr3^CE^-tdTomato^+^ cells (Fig. S10a–c). Concordantly, pRUNX2^+^ tdTomato-negative cells were present in the “bony bridge” of Fgfr3-Runx2^cKO^ synchondroses (Fig. S10d, yellow dashed lines), showing that RUNX2 functions in a cell population that is not derived from Fgfr3^+^ cells. These results confirm the efficiency of *Fgfr3-creER* to inactivate *Runx2* in Fgfr3^+^ cells. Thus, RUNX2 is critical in maintaining the organization of chondrocytes of the postnatal cranial base synchondrosis.

### *Runx2* inactivation impairs osteoblast differentiation of Fgfr3^+^ cells in the cranial base synchondroses

**Fig. 4:**
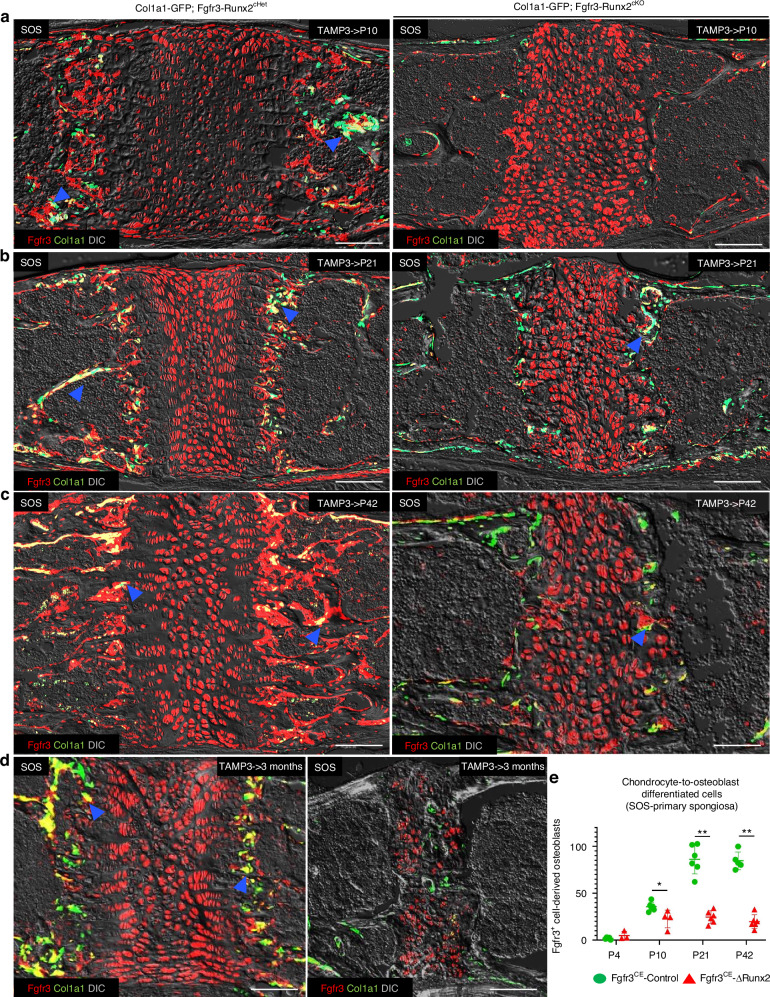
*Runx2* inactivation impairs osteoblast differentiation of Fgfr3^+^ cells in the synchondrosis. Lineage-tracing of Col1a1(2.3 kb)-GFP^+^Fgfr3^CE^-tdT^+^ SOS cells in Fgfr3-Runx2^cHet^ and Fgfr3-Runx2^cKO^ mice following tamoxifen injection at P3 and lineage-traced to P10 (**a**) P21 (**b**) P42 (**c**) and 3 months (**d**). Blue arrowheads indicate Col1a1(2.3 kb)-GFP^+^Fgfr3^CE^-tdT^+^ differentiated cells at the primary spongiosa. Red: Fgfr3^CE^-tdT, green: Col1a1(2.3 kb)-GFP, yellow: Col1a1(2.3 kb)-GFP^+^Fgfr3^CE^-tdT^+^, gray: DIC. Scale bar: 100 µm. **e** Quantification of Col1a1(2.3 kb)-GFP^+^Fgfr3^CE^-tdT^+^ cells at primary spongiosa at P4 (Fgfr3^CE^-Control *n* = 5, Fgfr3^CE^-∆Runx2 *n* = 3), P10 (Fgfr3^CE^-Control *n* = 6, Fgfr3^CE^-∆Runx2 *n* = 4), P21 (Fgfr3^CE^-Control n*n* = 6, Fgfr3^CE^-∆Runx2 *n* = 6) and P42 (Fgfr3^CE^-Control *n* = 5, Fgfr3^CE^-∆Runx2 *n* = 6) collected from serial sections of SOS at all time points. *P < 0.05, **P < 0.01, Mann-Whitney’s *U*-test. Data are presented as mean ± s.d

Subsequently, we asked whether Fgfr3^+^ cell fates are altered in the cranial base synchondrosis due to *Runx2* inactivation. To this end, we crossed Fgfr3-Runx2^cHet^ and Fgfr3-Runx2^cKO^ mice with *Col1a1(2.3* *kb)-GFP* and *Rosa26*-*tdTomato* reporter lines, allowing cell linage analyses. *Col1a1-GFP; Fgfr3-creER; Runx2*^*fl/+*^*; R26R*^*tdTomato*^ (Fgfr3-Runx2^cHet^) and *Col1a1-GFP; Fgfr3-creER; Runx2*^*fl/fl*^*; R26R*^*tdTomato*^ mice (Fgfr3-Runx2^cKO^) were injected with a single dose of tamoxifen at P3 and lineage-traced to follow *Runx2*-deficient tdTomato-labeled cells (Fgfr3^CE^-Control and Fgfr3^CE^-∆Runx2 cells). Histologically, trabecular bone formation was decreased in Fgfr3-Runx2^cKO^ synchondroses associated with a marked decrease in Col1a1(2.3 kb)-GFP^+^ cells (Fig. 4a–d, right panels and Fig. S11a–d, right panels). Moreover, chondrocyte layers were disorganized with altered cellular morphology in Fgfr3-Runx2^cKO^ synchondroses. Interestingly, at 3 months, the Col1a1-GFP^+^ osteoblasts occupied the “bony bridge” in the disorganized SOS. However, these cells were tdTomato negative, so they were not derived from Fgfr3^+^ cells. These osteoblasts mediating bony bridge formation must have come from a different yet-to-be-identified cellular source (Fig. 4d, right panel).

Fgfr3^CE^-∆Runx2^+^ chondrocytes and Col1a1-GFP^+^ osteoblasts in the primary spongiosa were significantly decreased in Fgfr3-Runx2^cKO^ synchondroses at P21 (SOS) and P42 (SOS and ISS) (Figs. S11f, g and S12a, b). Col1a1-GFP^+^Fgfr3^CE^-∆Runx2^+^ osteoblasts were also decreased in the primary spongiosa adjacent to the synchondroses at P10, P21, and P42 (Figs. 4e and S11e). Overall, *Runx2* inactivation in Fgfr3^+^ cells leads to the replacement of degraded SOS chondrocytes by osteoblasts emanating from a different cell of origin.

### *Runx2* inactivation impairs proliferation and promotes apoptosis of synchondrosis chondrocytes

**Fig. 5:**
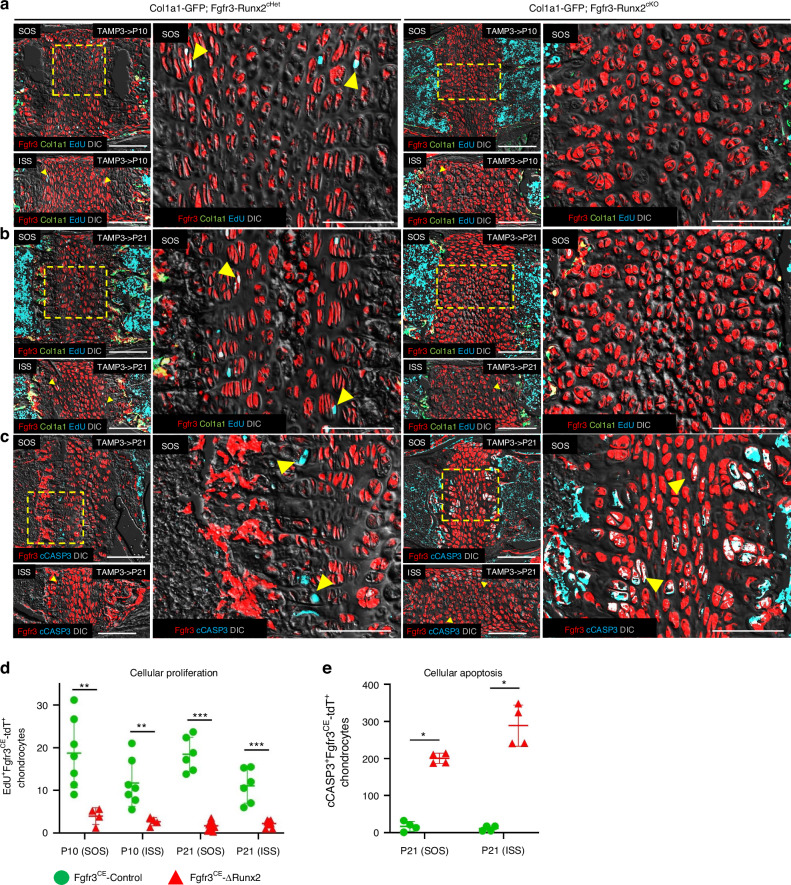
*Runx2* inactivation impairs proliferation and promotes apoptosis of synchondrosis chondrocytes. Fgfr3-Runx2^cHet^ and Fgfr3-Runx2^cKO^ mice were injected with tamoxifen at P3 and lineage-traced to P10 (**a**) and P21 (**b**). Mice were injected with EdU 3 h prior to euthanasia. Boxed regions show higher magnification. EdU^+^ Fgfr3^CE^-Control^+^ cells are present in the proliferating zone of Fgfr3-Runx2^cHet^ synchondroses at P10 and P21 (**a**, **b** left magnified panels, arrowheads). EdU^+^ cells populate Fgfr3^CE^-tdT^−^ bone marrow stromal cells throughout the primary spongiosa but do not label Fgfr3^CE^-∆Runx2^+^ synchondrosis chondrocytes (**a**, **b** right magnified panels). Red: Fgfr3^CE^-tdT, green: Col1a1(2.3 kb)-GFP, yellow: Col1a1(2.3 kb)-GFP; Fgfr3^CE^-tdT, blue: EdU, gray: DIC. **c** Fgfr3-Runx2^cHet^ and Fgfr3-Runx2^cKO^ mice were injected with tamoxifen at P3 and lineage-traced to P21. Synchondroses were stained with cCASP3. Boxed regions show higher magnification. cCASP3^+^Fgfr3^CE^-Control^+^ hypertrophic chondrocytes sparsely label Fgfr3-Runx2^cHet^ synchondroses (**c** left magnified panels, arrowheads). cCASP3^+^Fgfr3^CE^-∆Runx2^+^ robustly label all Fgfr3-Runx2^cKO^ synchondrosis chondrocytes (**c**, right magnified panels, arrowheads). Red: Fgfr3^CE^-tdT, green: Col1a1(2.3 kb)-GFP, yellow: Col1a1(2.3 kb)-GFP^+^Fgfr3^CE^-tdT^+^, blue: cCASP3, gray: DIC. Scale bar: 100 µm. **d** Quantification of EdU^+^Fgfr3^CE^-tdT^+^ chondrocytes at P10 (Fgfr3^CE^-Control *n* = 7, Fgfr3^CE^-∆Runx2 *n* = 5) and P21 [Fgfr3^CE^-Control *n* = 6, Fgfr3^CE^-∆Runx2 *n* = 9 (SOS)/*n* = 7 (ISS)]. **P < 0.01, ***P < 0.001, Mann-Whitney’s *U*-test. Data are presented as mean ± s.d. **e** Quantification of cCASP3^+^Fgfr3^CE^-tdT^+^ synchondrosis chondrocytes at P21 (Fgfr3^CE^-Control *n* = 4, Fgfr3^CE^-∆Runx2 *n* = 4). *P < 0.05, Mann-Whitney’s *U*-test. Data are presented as mean ± s.d

Next, we investigated the cellular basis of impaired anterior-posterior skull growth observed in Fgfr3-Runx2^cKO^ mice. To this end, we examined the proliferation and apoptosis of synchondrosis chondrocytes. Fgfr3-Runx2^cHet^ and Fgfr3-Runx2^cKO^ mice were analyzed at P10 and P21. Mice were injected with EdU 3 hours prior to euthanasia. Actively proliferating cells that incorporated EdU were fluorescently marked by Alexa Flour 647. As expected, EdU^+^Fgfr3^CE^-Control^+^ cells were localized to the proliferating zone (Fig. 5a, b, left panels, arrowheads). However, EdU^+^ cells were nearly absent in Fgfr3-Runx2^cKO^ synchondroses (Fig. 5a, b, right panels). Notably, seemingly equivalent numbers of EdU^+^ cells were observed in the bone marrow of both Fgfr3-Runx2^cHet^ and Fgfr3-Runx2^cKO^ synchondroses (Fig. 5a, b). Quantification of EdU^+^ cells revealed that RUNX2 deficiency inhibited the proliferation of synchondrosis chondrocytes (Fig. 5d). We next evaluated cellular apoptosis in Fgfr3-Runx2^cHet^ and Fgfr3-Runx2^cKO^ synchondroses at P21 by measuring protein levels of cleaved Caspase 3 (cCASP3), a marker of chondrocyte apoptosis associated with extracellular matrix degradation.^43^ cCASP3^+^Fgfr3^CE^-Control^+^ cells were localized to the hypertrophic zone adjacent to the primary spongiosa (Fig. 5c, left panels, arrowheads). In contrast, cCASP3^+^Fgfr3^CE^-∆Runx2^+^ cells were dramatically increased, with apoptotic cells exhibiting a random distribution throughout Fgfr3-Runx2^cKO^ synchondroses (Fig. 5c, right panels; e). Thus, *Runx2* inactivation suppresses proliferation and stimulates apoptosis of synchondrosis chondrocytes.

### *Runx2* inactivation causes precocious hypertrophy of synchondrosis chondrocytes

**Fig. 6:**
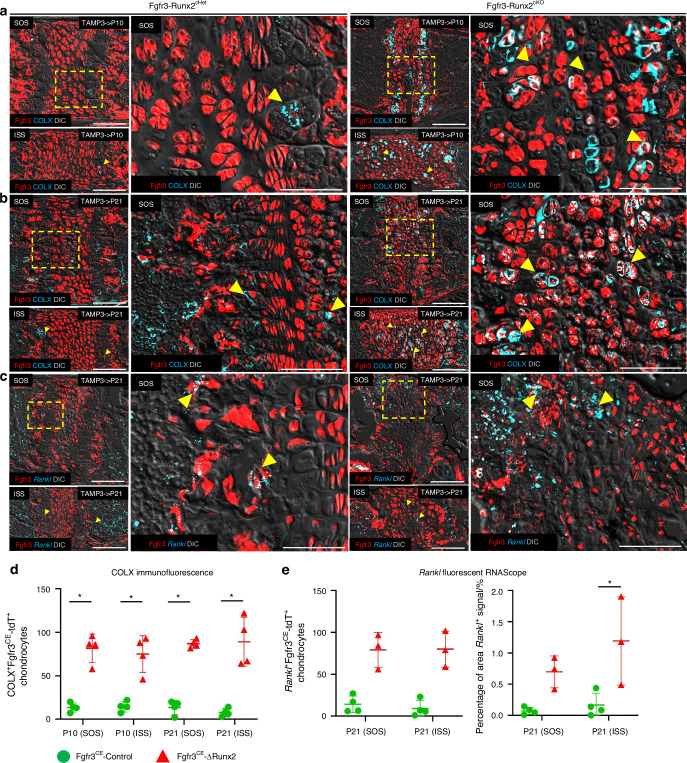
*Runx2* inactivation causes precocious hypertrophy of synchondrosis chondrocytes. Fgfr3-Runx2^cHet^ and Fgfr3-Runx2^cKO^ mice were injected with tamoxifen at P3 and lineage-traced to P10 (**a**) and P21 (**b**). Synchondroses were stained with COLX. Boxed regions show higher magnification. COLX^+^Fgfr3^CE^-Control^+^ cells sparsely label Fgfr3-Runx2^cHet^ hypertrophic chondrocytes (**a**, **b** left magnified panels, arrowheads). COLX^+^Fgfr3^CE^-∆Runx2^+^ synchondrosis chondrocytes robustly label Fgfr3-Runx2^cKO^ synchondroses (**a**, **b**, right magnified panels, arrowheads). Red: Fgfr3^CE^-tdT, blue: COLX, gray: DIC. Scale bar: 100 µm. **c** Fgfr3-Runx2^cHet^ and Fgfr3-Runx2^cKO^ mice were injected with tamoxifen at P3 and lineage-traced to P10 (**a**) and P21 (**b**). Synchondroses were stained with *Rankl*. Boxed regions show higher magnification. *Rankl*^+^Fgfr3^CE^-Control^+^ cells sparsely label Fgfr3-Runx2^cHet^ hypertrophic chondrocytes (**a**, **b** left magnified panels, arrowheads). *Rankl*^+^Fgfr3^CE^-∆Runx2^+^ synchondrosis chondrocytes robustly label Fgfr3-Runx2^cKO^ synchondroses (**a**, **b** right magnified panels, arrowheads). Red: Fgfr3-CE^tdT^, blue: *Rankl*, gray: DIC. Scale bar: 100 µm. **d** Quantification of COLX^+^Fgfr3^CE^-tdT^+^ synchondrosis chondrocytes at P10 (Fgfr3^CE^-Control *n* = 4, Fgfr3^CE^-∆Runx2 *n* = 4) and P21 (Fgfr3^CE^-Control *n* = 4, Fgfr3^CE^-∆Runx2 *n* = 4). *P < 0.05, Mann-Whitney’s *U*-test. Data are presented as mean ± s.d. **e** Quantification of *Rankl*^+^Fgfr3^CE^-tdT^+^ synchondrosis chondrocytes (left graph, Fgfr3^CE^-Control *n* = 4, Fgfr3^CE^-∆Runx2 *n* = 3) and % area *Rankl*^+^ signal (right graph, Fgfr3^CE^-Control *n* = 4, Fgfr3^CE^-∆Runx2 *n* = 3) at P21. *P < 0.05, Mann-Whitney’s *U*-test. Data are presented as mean ± s.d

As noted above, cells resembling hypertrophic chondrocytes are dispersed throughout Fgfr3-Runx2^cKO^ synchondroses. To define the properties of these cells, we measured levels of type X collagen (COLX), a marker of hypertrophic chondrocytes, in Fgfr3-Runx2^cHet^ and Fgfr3-Runx2^cKO^ synchondroses using immunohistochemistry. COLX^+^Fgfr3^CE^-Control^+^ cells were localized to the hypertrophic zone adjacent to the primary spongiosa and central zone at P10, P21 (Fig. 6a, b, left magnified panels, arrowheads), P42, and 3 months (Fig. S13a, c, left magnified panels, arrowheads). The identity of these COLX^+^ cells as hypertrophic chondrocytes was confirmed by H&E staining (Fig. S13b, d, left magnified panels, arrowheads). In contrast, COLX^+^Fgfr3^CE^-∆Runx2^+^ cells were increased and randomly distributed throughout Fgfr3-Runx2^cKO^ synchondroses at P10 and P21 (Fig. 6a, b, right panels; d). At P42 and 3 months, COLX^+^ cells were slightly increased in the Fgfr3^CE^-∆Runx2^+^ synchondrosis, localized to the border with the adjacent primary spongiosa (Fig. S13a, c, right magnified panels, arrowheads), as confirmed by H&E staining (Fig. S13b, d, right magnified panels, arrowheads). Since hypertrophic chondrocytes support osteoclastogenesis via RANKL expression,^44^ we measured *Tnfrsf11a* (*Rankl*) mRNA levels using fluorescent RNAScope assays. *Rankl* mRNA expression was dramatically elevated and widely distributed throughout Fgfr3-Runx2^cKO^ synchondroses (Fig. 6c, right panels, arrowheads). The numbers of *Rankl*^+^Fgfr3^CE^-∆Runx2^+^ cells and the total *Rankl*^+^ signals were increased in Fgfr3-Runx2^cKO^ synchondroses (Fig. 6e). Thus, the premature hypertrophy of Fgfr3^CE^-∆Runx2^+^ chondrocytes is associated with increased *Rankl* expression. This finding compelled us to investigate the role of osteoclastogenesis in the degradation of Fgfr3-Runx2^cKO^ synchondroses.

### Osteoclast-driven degradation of Fgfr3-Runx2^cKO^ synchondroses

**Fig. 7:**
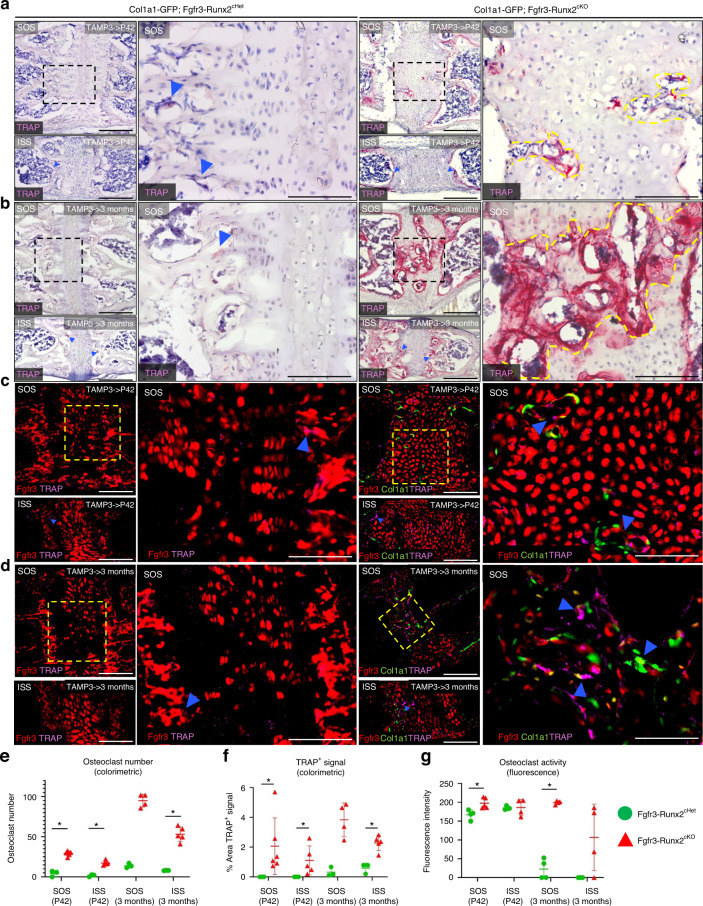
Osteoclast-driven degradation of Fgfr3-Runx2^cKO^ synchondroses. Fgfr3-Runx2^cHet^ and Fgfr3-Runx2^cKO^ mice were injected with tamoxifen at P3 and lineage-traced to P42 (**a**) and 3 months (**b**). Synchondroses were stained with tartrate-resistant acid phosphatase (TRAP). Boxed regions show higher magnification. TRAP^+^ osteoclasts sparsely label the primary spongiosa at P42 and 3 months in Fgfr3-Runx2^cHet^ synchondroses (**a**, **b** left magnified panels, arrowheads). TRAP^+^ signal envelopes the entire fusion defect in the SOS of Fgfr3-Runx2^cKO^ animals (**a**, **b** right magnified panels, yellow dashed lines). Fgfr3-Runx2^cHet^ and Fgfr3-Runx2^cKO^ mice were injected with tamoxifen at P3 and lineage-traced to P42 (**c**) and 3 months (**d**). Synchondroses were stained with fluorescent TRAP (ELF97). Boxed regions show higher magnification. TRAP^+^ osteoclasts sparsely label the primary spongiosa at P42 and 3 months in Fgfr3-Runx2^cHet^ synchondroses (**c**, **d** left magnified panels, arrowheads). In the SOS of Fgfr3-Runx2^cKO^ mice, Col1a1(2.3 kb)-GFP^+^, Fgfr3^CE^-tdT^+^, TRAP^+^ cells independently label the fusion defect (**c**, **d** right magnified panels, arrowheads). Red: Fgfr3^CE^-tdT, green: Col1a1(2.3 kb)-GFP, purple: TRAP. Scale bar: 100 µm. Quantification of colorimetric TRAP^+^ osteoclasts (**e**) and % area TRAP^+^ signal (**f**) at P42 [Fgfr3-Runx2^cHet^ *n* = 3, Fgfr3-Runx2^cKO^ *n* = 6 (SOS)/*n* = 5 (ISS)] and 3 months [Fgfr3-Runx2^cHet^ *n* = 3, Fgfr3-Runx2^cKO^ *n* = 4 (SOS)/*n* = 5 (ISS)]. *P < 0.05, Mann-Whitney’s *U*-test. Data are presented as mean ± s.d. **g** Quantification of fluorescent TRAP^+^ osteoclasts at P42 [Fgfr3-Runx2^cHet^ *n* = 4, Fgfr3-Runx2^cKO^ *n* = 5 (SOS)/*n* = 4 (ISS)] and 3 months [Fgfr3-Runx2^cHet^ *n* = 4 (SOS)/*n* = 3 (ISS), Fgfr3-Runx2^cKO^ *n* = 4 (SOS)/*n* = 4 (ISS)]. *P < 0.05, Mann-Whitney’s *U*-test. Data are presented as mean ± s.d

To examine osteoclastogenesis, we performed tartrate-resistant acid phosphatase (TRAP) staining on Fgfr3-Runx2^cHet^ and Fgfr3-Runx2^cKO^ synchondroses using both colorimetric (Fig. 7a, b) and fluorescent protocols (Fig. 7c, d). TRAP^+^ osteoclasts were present in the degraded regions of Fgfr3-Runx2^cKO^ mouse SOS at P42 (Fig. 7A, right panels, yellow dashed lines) and 3 months (Fig. 7b, right panels, yellow dashed lines). Analysis of fluorescent TRAP staining using ELF97 fluorescent substrate demonstrated that osteoclasts in the prematurely ossified SOS of Fgfr3-Runx2^cKO^ mice were adjacent to both Fgfr3^CE^-∆Runx2^+^ chondrocytes and Col1a1(2.3 kb)-GFP^+^ osteoblasts (Fig. 7c, d, right panels, arrowheads). Interestingly, a similar ossification defect was not observed in the ISS of these mice, although increased TRAP staining was still seen via the colorimetric assay (Fig. 7a, b, right lower panels). The numbers of TRAP^+^ osteoclasts and total percentages of TRAP^+^ signal were increased in Fgfr3-Runx2^cKO^ synchondroses (Fig. 7e, f). Notably, ELF97^+^ fluorescence intensity, which measures osteoclast activity, was increased in the SOS of Fgfr3-Runx2^cKO^ animals at P42 and 3 months (Fig. 7g). Given the established role of hypertrophic chondrocytes in angiogenesis,^45^ we also defined CD31 and VEGFR expressions in the Fgfr3-Runx2^cHet^ and Fgfr3-Runx2^cKO^ synchondroses.^46,47^ Robust CD31 and VEGFR immunohistochemical signals were observed in the Fgfr3-Runx2^cHet^ synchondroses (Fig. S14a, b, left magnified panels). In contrast, CD31 and VEGFR signals were markedly decreased in the Fgfr3-Runx2^cKO^ synchondroses, particularly localized to Fgfr3^CE^-tdT^−^ areas associated with “bony bridge” formation (Fig. S14a, b, right magnified panels, arrowheads). Thus, *Runx2* inactivation in synchondrosis chondrocytes enhanced osteoclastogenesis and reduced angiogenesis, resulting in the premature degradation of synchondrosis and eventual fusion.

### RUNX2-FGFR3-MAPK-SOX9 signaling controls postnatal cranial base development

Our findings thus far prompted us to examine cell-intrinsic molecular changes in *Runx2*-deficient chondrocytes that drive premature fusion of the cranial base synchondrosis. To provide a potential explanation for these results, we considered the possibility that RUNX2 may control synchondrosis hypertrophy by negatively regulating FGFR3.

**Fig. 8:**
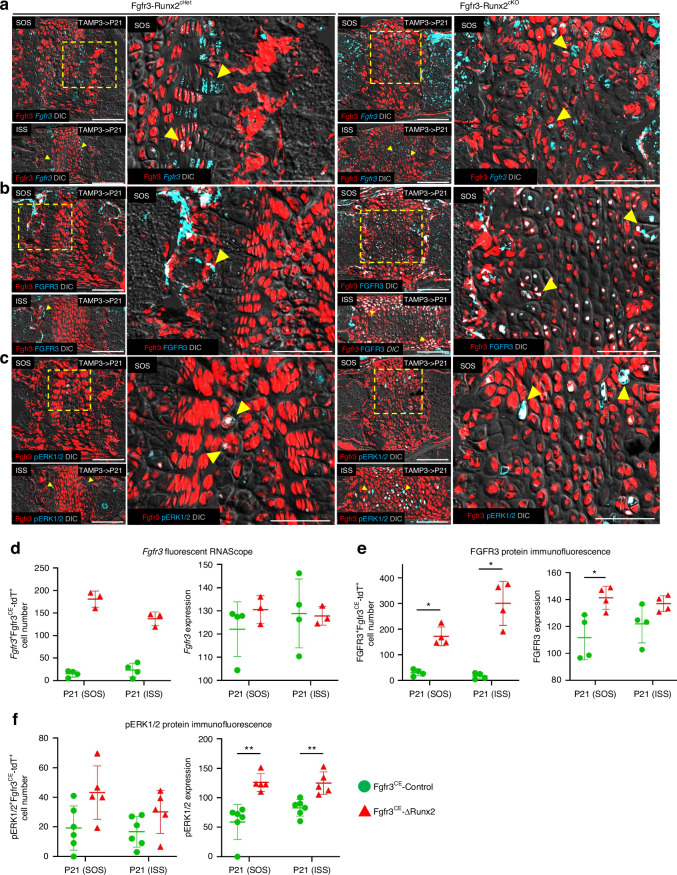
RUNX2-FGFR3-MAPK-SOX9 signaling controls postnatal cranial base development. Fgfr3-Runx2^cHet^ and Fgfr3-Runx2^cKO^ mice were injected with tamoxifen at P3 and lineage-traced to P21. Synchondroses were stained with *Fgfr3* (**a**) and FGFR3 (**b**). Boxed regions show higher magnification. *Fgfr3*^+^Fgfr3^CE^-Control^+^ and FGFR3^+^Fgfr3^CE^-Control^+^ expression is localized to proliferating and pre-hypertrophic chondrocytes, respectively, in Fgfr3-Runx2^cHet^ synchondroses (**a**, **b**, left magnified panels, arrowheads). *Fgfr3*^+^Fgfr3^CE^-∆Runx2^+^ cells are distributed randomly throughout *Runx2*^*cKO*^ synchondroses (**a**, right magnified panels, arrowheads). FGFR3^+^Fgfr3^CE^-∆Runx2 chondrocytes robustly label chondrocyte layers in Fgfr3-Runx2^cKO^ synchondroses (**b**, right magnified panels, arrowheads). Red: Fgfr3^CE^-tdT, blue: *Fgfr3* (**a**) or FGFR3 (**b**), gray: DIC. Scale bar: 100 µm. **c** Fgfr3-Runx2^cHet^ and Fgfr3-Runx2^cKO^ mice were injected with tamoxifen at P3 and lineage-traced to P21. Synchondroses were stained with pERK1/2. Boxed regions show higher magnification. pERK1/2^+^Fgfr3^CE^-Control^+^ cells sparsely label Fgfr3-Runx2^cHet^ hypertrophic-like chondrocytes at low levels (**c**, left magnified panels, arrowheads). pERK1/2^+^Fgfr3^CE^-∆Runx2^+^ synchondrosis chondrocytes label Fgfr3-Runx2^cKO^ synchondroses at greater numbers and increased fluorescence intensities (**a**, **b**, right magnified panels, arrowheads). Red: Fgfr3^CE^-tdT, blue: pERK1/2, gray: DIC. Scale bar: 100 µm. **d** Quantification of *Fgfr3*^+^Fgfr3^CE^-tdT^+^ synchondrosis chondrocytes (left graph) and *Fgfr3*^+^ fluorescence intensity (right graph) at P21 (Fgfr3^CE^-Control *n* = 4, Fgfr3^CE^-∆Runx2 *n* = 3). Mann-Whitney’s *U*-test. Data are presented as mean ± s.d. **e** Quantification of FGFR3^+^Fgfr3^CE^-tdT^+^ synchondrosis chondrocytes (left graph) and FGFR3^+^ fluorescence intensity (right graph) at P21 (Fgfr3^CE^-Control *n* = 4, Fgfr3^CE^-∆Runx2 *n* = 4). *P < 0.05, Mann-Whitney’s *U*-test. Data are presented as mean ± s.d. **f** Quantification of pERK1/2^+^Fgfr3^CE^-tdT^+^ synchondrosis chondrocytes (left graph) and pERK1/2^+^ fluorescence intensity (right graph) at P21 (Fgfr3^CE^-Control *n* = 6, Fgfr3^CE^-∆Runx2 *n* = 5). **P < 0.01, Mann-Whitney’s *U*-test. Data are presented as mean ± s.d

This focus on FGFR3 is based on two observations. First, the premature fusion of the cranial base synchondroses observed in Fgfr3-Runx2^cKO^ mice is reminiscent of the phenotype of mice harboring *Fgfr3* activating mutations where similar defects in anterior-posterior cranial growth were observed.^35^ Second, prior cell culture studies suggested that RUNX2 can directly interact with the *Fgfr3* promoter to regulate its gene expression.^37^ We, therefore, investigated alterations of *Fgfr3* mRNA and FGFR3 protein at P21 in Fgfr3-Runx2^cHet^ and Fgfr3-Runx2^cKO^ synchondrosis chondrocytes using fluorescent RNAscope and immunofluorescence assays. In Fgfr3-Runx2^cHet^ synchondroses, *Fgfr3* mRNA was localized in proliferating chondrocytes (Fig. 8a, left panels, arrowheads). In contrast, in Fgfr3-Runx2^cKO^ synchondroses, its distribution expanded to hypertrophic chondrocyte-like cells within the central portion of the synchondroses (Fig. 8a, right panels, arrowheads). Quantification of *Fgfr3*-expressing Fgfr3^CE^-∆Runx2^+^ cells confirmed an increase in both the SOS and ISS of Fgfr3-Runx2^cKO^ mice (Fig. 8d, left). FGFR3 protein exhibited localized expression in a small population of pre-hypertrophic and hypertrophic cells in Fgfr3-Runx2^cHet^ synchondroses (Fig. 8b, left panels, arrowheads). Conversely, it was robustly expressed throughout multiple layers of Fgfr3-Runx2^cKO^ synchondroses, primarily in an intranuclear fashion (Fig. 8b, right panels, arrowheads), as reflected by an increase in FGFR3-expressing Fgfr3^CE^-∆Runx2^+^ chondrocytes (Fig. 8e, left). Notably, FGFR3 fluorescence intensity, an indicator of protein abundance, was increased in the SOS of Fgfr3-Runx2^cKO^ mice (Fig. 8e, right), suggesting that FGFR3 levels may be elevated in synchondrosis chondrocytes in the absence of *Runx2*, leading to aberrant downstream signaling.

To further evaluate this aspect, protein levels of phospho-ERK1/2 (pERK1/2), a downstream effector of FGFR3 in chondrocytes,^48^ were evaluated in Fgfr3-Runx2^cHet^ and Fgfr3-Runx2^cKO^ synchondroses at P21. A few pERK1/2-expressing Fgfr3^CE^-Control^+^ hypertrophic chondrocytes were present in Fgfr3-Runx2^cHet^ synchondroses (Fig. 8c, left panels, arrowheads). In contrast, pERK1/2-expressing Fgfr3^CE^-∆Runx2^+^ hypertrophic chondrocytes were moderately increased and randomly distributed in Fgfr3-Runx2^cKO^ synchondroses (Fig. 8c, right panels, arrowheads, and Fig. 8f, left). Notably, the total cell fluorescence intensity of pERK1/2 was increased in Fgfr3^CE^-∆Runx2^+^ chondrocytes (Fig. 8f, right), suggesting that FGFR3 downstream signaling is elevated in response to *Runx2* inactivation in synchondrosis chondrocytes.

SOX9 is a second FGFR3 downstream target directly activated by ERK signaling via upstream FGFR3 activity in chondrocytes.^49^ Consequently, we evaluated SOX9 expression in Fgfr3^CE^-Control^+^ and Fgfr3^CE^-∆Runx2^+^ synchondrosis chondrocytes at P21. While we observed a similar distribution of SOX9 protein through all chondrocyte layers in both groups (Fig. S15a, b, left), the fluorescence intensity of SOX9 protein staining was increased in Fgfr3^CE^-∆Runx2^+^ chondrocytes (Fig. S15b, right).

Collectively, our results suggest that *Runx2* inactivation in cranial base synchondroses may stimulate FGFR3-MAPK-SOX9 signaling, leading to premature fusion of the postnatal cranial base synchondroses and subsequent defects in cranial growth.

## Discussion

Here, we report a novel role of RUNX2 in maintaining cartilage in the postnatal cranial base synchondroses. Loss of *Runx2* in synchondrosis chondrocytes leads to disruption of the well-defined layers of resting, proliferating, and hypertrophic chondrocytes in the synchondrosis, associated with a decrease in proliferation and an increase in chondrocyte apoptosis. These changes are accompanied by inhibition of osteoblast differentiation, random distribution of hypertrophic chondrocyte-like cells throughout the synchondrosis, increased *Rankl* production, and subsequent osteoclastogenesis, leading to premature synchondrosis degradation and eventual fusion. *Runx2* loss also causes increases in FGFR3 and related downstream signaling pathways, suggesting that phenotypic alterations in mutant synchondroses may be driven by excessive FGFR3, whose activation is known to inhibit chondrocyte proliferation, leading to premature hypertrophy and synchondrosis fusion.

We recently reported that *Fgfr3-creER* marks endosteal stromal cells in postnatal long bones.^39^ The present study focused on the distribution of Fgfr3^+^ cells and their descendants in the cranial base synchondroses. As shown, *Fgfr3-creER* robustly labels synchondrosis chondrocytes upon tamoxifen injection following short (P4) and long (8 months) lineage-tracing experiments, suggesting that Fgfr3^CE^-tdT^+^ cells contribute both to synchondrosis organization and long-term tissue maintenance. Significantly, Fgfr3^CE^-tdT^+^ cells are shown to differentiate into osteoblasts at the primary spongiosa, which is similar to their long bone counterparts. Moreover, we observed a moderate increase in Col1a1-GFP^+^ osteoblasts in the SOS, but not in the ISS, from P4 to P42 possibly due to the late timing that the SOS ossifies postnatally. These studies also identify *Fgfr3-creER* as a reliable tool for studying gene function in synchondrosis chondrocytes.

The master osteoblast regulatory gene, *Runx2*, is required for skeletal development^50^ and functions in the craniofacial region. *RUNX2* haploinsufficiency in humans and mice causes cleidocranial dysplasia associated with midface hypoplasia related to reduced anterior-posterior skull growth and open posterior frontal and sagittal cranial sutures. In mice, sutural changes have been attributed to aberrant transcriptional regulation of the Hedgehog, FGF, Msx2, and Wnt signaling pathways.^28^ Although RUNX2 is expressed in the cranial base anlage,^27^ until the present study, its function in the synchondroses remained unknown. We conditionally deleted *Runx2* in postnatal synchondrosis chondrocytes using *Fgfr3-creER* and found that Fgfr3-Runx2^cKO^ mice displayed reduced anteroposterior skull growth, widened cranial vaults, and premature synchondrosis ossification. Fgfr3-Runx2^cKO^ mice also displayed skeletal dwarfism, as evidenced by reductions in body weight, naso-anal, and tail lengths. The ratios of anterior cranial vault width to facial height and middle cranial vault width to height were increased while the facial length to anterior cranial vault width, cranial vault length to height, and cranial vault length to middle vault width were decreased in Fgfr3-Runx2^cKO^ mice. Note that cranial vault shape was mildly altered in a subgroup of Fgfr3-Runx2^cHet^ mice at P9, but these differences from wild-type mice disappeared by P42 in both sexes. These observations highlight a possible catch-up growth to compensate for mild craniofacial deformations due to the loss of one functional *Runx2* allele in Fgfr3^+^ cells. These results demonstrate that overall craniofacial proportions are disrupted in Fgfr3-Runx2^cKO^ mice, including increases in cranial vault width and decreases in facial and cranial vault lengths.

Examination of 3D-rendered cranial vaults of Fgfr3-Runx2^cKO^ mice showed intermittent mild premature fusion of the coronal suture in Fgfr3-Runx2^cKO^ male mice. Conversely, RUNX2 overexpression in Prrx1^+^ mesenchymal cells has been shown to cause premature suture fusion during embryonic development.^51^ Therefore, RUNX2 may regulate the suture patency context dependently, with various factors such as the cell type and sex modulating its function. In humans, non-syndromic craniosynostoses affect males more frequently than females,^52^ consistent with our findings. Collectively, our results point to the possible contribution of synchondrosis RUNX2 to maintaining suture patency in postnatal development.

In long bone growth plates, *Runx2* deletion in chondrocytes impairs endochondral ossification^53,54^ and prevents differentiation of growth plate hypertrophic chondrocytes to osteoblasts.^55,56^ Our results support an even more prominent function of RUNX2 in synchondroses. In Fgfr3-Runx2^cKO^ synchondroses, we found a striking reduction in Fgfr3^+^ cell-derived osteoblasts in the primary spongiosa and chondrocytes in the synchondrosis at P42. Importantly, we also observed a decrease in non-Fgfr3^+^ cell-derived osteoblasts at the primary spongiosa in the Fgfr3-Runx2^cKO^ synchondrosis. Thus, without RUNX2, defects appear in both chondrocyte-dependent and chondrocyte-independent osteoblast differentiation. However, as we cannot reliably distinguish two processes with our current methodology, the contribution of each respective process to the observed phenotype remains undefined. In the absence of RUNX2 in Fgfr3^+^ cells, we also noted that pRUNX2^*+*^ osteoblasts arise from a separate (Fgfr3-negative) cellular source that partially accounts for premature synchondrosis fusion. Importantly, phenotypic differences were noted among the SOS, ISS, and AIOS in Fgfr3-Runx2^cKO^ mice. Although the organization of all three synchondroses was disrupted, “bony bridge” formation was only seen in the SOS (Figs. 3 and S9). This may be explained by the differences in the maturation and fusion rates of different synchondroses or their distinct embryonic origins (neural crest or mesoderm-derived). Further studies are warranted to determine if a similar ossification defect arises in the ISS or AIOS if we broaden the timeline of our studies.

Chondrocyte-selective inactivation of *Runx2* in synchondroses significantly reduced cell proliferation, associated with a dramatic increase in apoptosis. Prior studies examining the effects of *Runx2* deletion in long bone growth plate chondrocytes also reported reduced chondrocyte proliferation, leading to stunted limb growth.^56^ However, inhibition of apoptosis was also reported. The basis for these differences needs to be clarified, but could be related to the different cells targeted for *Runx2* inactivation (Fgfr3^+^ chondrocytes in our study versus COLX^+^ hypertrophic chondrocytes in the study by Rashid, Ghori-Javed et al.^57^) or reflect intrinsic differences between synchondroses and long bone growth plates.

Chondrocyte hypertrophy, which is associated with cellular swelling,^58,59^ was evaluated in Fgfr3-Runx2^cKO^ synchondroses by measuring COLX, a marker for hypertrophic cells that RUNX2 regulates.^60–62^ Surprisingly, we found that COLX^+^ hypertrophic chondrocytes dramatically increased and were randomly distributed in Fgfr3-Runx2^cKO^ synchondroses at P10 and P21. At P42 and 3 months, although we observed increased COLX^+^Fgfr3^CE^-tdT^+^ cells in the Fgfr3-Runx2^cKO^ synchondroses, these cells were mostly localized to the border with the primary spongiosa associated with areas of eventual “bony bridge” formation. These phenotypes may be induced by Fgfr3^CE^-∆Runx2^+^ chondrocytes progressing down their lineage, undergoing apoptosis, and being replaced by osteoblasts derived from non-chondrocyte sources. These results differ from previous findings in long bone growth plates where *Runx2* global knockout inhibited endochondral ossification and chondrocyte hypertrophy during late fetal development.^63^ Our result also contrasts with gain-of-function studies where RUNX2 overexpression in Col2a1^*+*^ chondrocytes causes premature COLX expression in vivo.^64^ RUNX2 overexpression in ATDC5 chondrogenic cells also increased COLX.^65,66^ These disparate results may reflect inherent differences between synchondrosis and growth plate chondrocytes or be related to the different cell populations where RUNX2 expression was altered (Fgfr3^+^ cells in our study versus global *Runx2* inactivation or overexpression in Col2a1^+^ cells in prior studies). Alternatively, chondrocyte hypertrophy may be directly or indirectly regulated by unknown mechanisms independent of RUNX2 under certain conditions. In any case, Fgfr3^CE^-∆Runx2^+^ synchondrosis chondrocytes undergo premature disorganized hypertrophy associated with elevated apoptosis.

Growth plate hypertrophic chondrocytes express RANKL, which stimulates osteoclastogenesis.^65,67^ *Rankl* deletion in hypertrophic chondrocytes causes osteopetrosis due to reduced cartilage resorption.^68^ Thus, hypertrophic chondrocyte-mediated RANKL secretion facilitates chondrocyte-to-osteoblast transformation by removal of nascent cartilage, allowing new bone formation. We discovered that total *Rankl* mRNA expression was increased in Fgfr3-Runx2^cKO^ synchondroses at P21, which we believe relates to increased hypertrophic chondrocytes. We next asked whether enhanced osteoclastogenesis contributes to the degradation of the Fgfr3-Runx2^cKO^ SOS. Robust osteoclast activity was observed in the SOS and ISS of Fgfr3-Runx2^cKO^ mice, although bony bridge formation and premature synchondrosis fusion were only observed in the SOS. The basis for differences between the SOS and ISS is unknown; however, the different embryologic origins of these two synchondroses and their corresponding ossification rates may contribute to this.^7^ Moreover, we observed reduced angiogenesis, as evaluated by CD31 and VEGFR expression, at 3 months in the Fgfr3-Runx2^cKO^ SOS localized to areas of putative “bony bridge” formation. Although hypertrophic chondrocytes stimulate angiogenesis via the expression of VEGFR, previous studies have also linked osteogenesis to angiogenesis via angiocrine mechanisms.^69^ Moreover, COLX expression in Fgfr3-Runx2^cHet^ and Fgfr3-Runx2^cKO^ synchondroses at 3 months were comparable. Thus, the reduction in Col1a1-GFP^+^ osteoblasts and osteocytes available in the Fgfr3-Runx2^cKO^ synchondroses at 3 months may reduce angiogenesis. In the *Runx2*-deficient SOS, chondrocytes undergo premature hypertrophy, stimulating aberrant osteoclastogenesis accompanied by localized angiogenesis and invasion of the resorption site with new bone that is formed in part by RUNX2^+^ osteoblasts that are not derived from Fgfr3^+^ cells.

Finally, we investigated the mechanisms through which RUNX2 signals control synchondrosis chondrocyte differentiation. We focused on FGFR3 based on similarities between the synchondrosis phenotype of Fgfr3-Runx2^cKO^ mice and that observed in mice and humans harboring FGFR3 activating mutations as well as in vitro evidence suggesting possible regulation of FGFR3 by RUNX2.^33,35,37,70,71^ Given the similarities in phenotypes in overactive FGFR3 and Fgfr3-Runx2^cKO^ animals, we investigated whether a RUNX2-FGFR3 mechanism might regulate synchondrosis development. In Fgfr3-Runx2^cKO^ synchondroses, we observed an increase in *Fgfr3* mRNA and FGFR3 protein expression. Interestingly, most FGFR3 was localized within the nucleus, similar to the FGFR3 distribution associated with *Fgfr3* activating mutations causing achondroplasia.^72^ Moreover, nuclear translocation of FGFR3 protein has also been tied to aberrant proliferation in breast and prostate cancers.^73,74^ Thus, RUNX2 deficiency in synchondrosis chondrocytes leads to elevated FGFR3 and possible receptor nuclear translocation, which may explain subsequent aberrant endochondral ossification and premature synchondrosis fusion.

Further evidence for increased FGFR3 signaling in Fgfr3-Runx2^cKO^ synchondroses was obtained when we examined the downstream proteins within the FGFR3 signaling pathway. FGFR3-MAPK signaling controls long bone growth plate development.^48,75^ Consistent with our findings, deletion of *Erk1/2* in growth plate chondrocytes increases cell proliferation,^28^ and *Erk1/*2 deletion in mesenchymal cells reduces RANKL expression and osteoclastogenesis.^76^ Also, constitutive activation of the FGFR3 effector, MEK1, which phosphorylates ERK1/2, in chondrocytes causes premature fusion of synchondroses.^46^ Further, complementary to our findings relating RUNX2 to FGFR3 signaling, suppressing pERK1/2 by meclozine dihydrochloride increases bone growth in achondroplasia mice.^77,78^ Our analyses indicate robust increases in pERK1/2 levels in Fgfr3^CE^-∆Runx2^+^ chondrocytes, confirming the possible activation of FGFR3 signaling via MAPK. We further evaluated SOX9 expression, a key regulator of chondrocyte formation.^79^ FGFR3 activation stimulates SOX9-mediated regulation of a *Col2a1* enhancer in primary chondrocytes via pERK1/2,^36^ and Sox9-binding sites are present within the *Fgfr3* promoter.^80^ Significantly, SOX9 represses COLX expression in resting and proliferating chondrocytes in vivo, restricting its activity to hypertrophic cells.^81^ We observed increases in SOX9 levels in *Runx2*^*cKO*^ synchondroses, suggesting that FGFR3-dependent pERK1/2 activation may lead to increased SOX9 activity. Notably, our findings are at odds with evidence showing that *Sox9* inactivation in growth plate chondrocytes causes disorganized growth plates associated with decreased *Col10a1* expression.^82^ Moreover, overexpression of *Sox9* in Col10^+^ hypertrophic and growth plate chondrocytes causes impaired skeletal growth due to reduced vascular invasion and osteoclastogenesis associated with an elevated *Col10a1* expression^83^ and reduced hypertrophic chondrocyte-to-osteoblast transdifferentiation associated with elevated chondrocyte apoptosis,^84^ respectively. Thus, although our findings point to a possible function of SOX9 in promoting *ColX* transcription due to overactive upstream FGFR3 signaling, specifically in synchondrosis chondrocytes, further studies are warranted to clarify SOX9 function as a downstream response to RUNX2 and FGFR3.

**Fig. 9:**
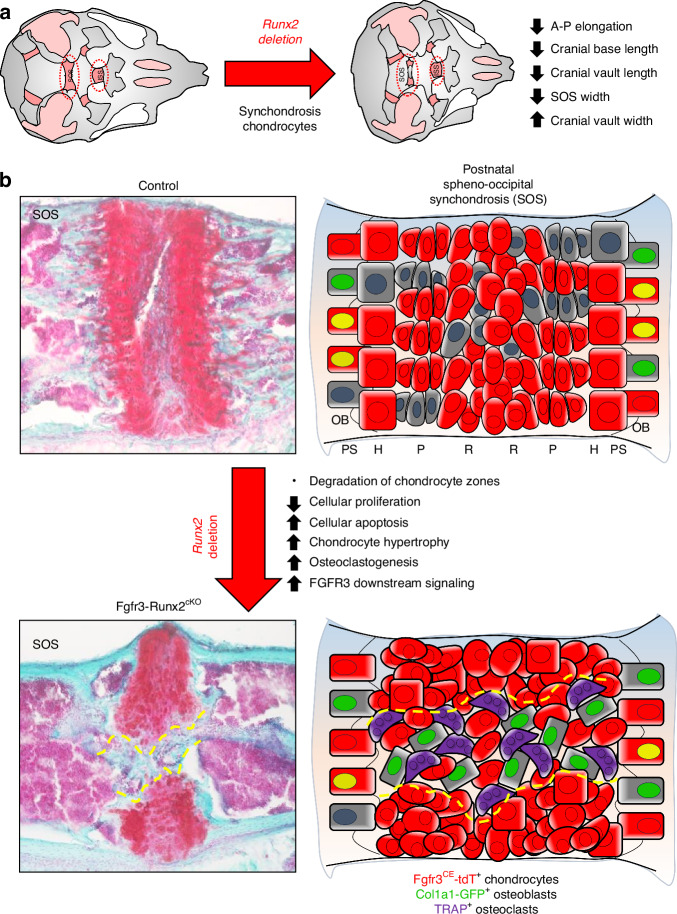
RUNX2 is a critical regulator of cranial base synchondrosis organization, maintenance, and fusion. **a** Postnatal *Runx2* deletion causes aberrant development of the craniofacial complex, leading to reductions in anteroposterior elongation, cranial base and vault lengths, and skull width. **b** *Runx2* deletion in synchondrosis chondrocytes causes degradation and premature ossification of the postnatal cranial base synchondroses via reduced cell proliferation, increased chondrocyte hypertrophy and death, osteoclastogenesis associated with localized angiogenesis and FGFR3 downstream signaling, leading to aberrant endochondral ossification and gross craniofacial structural deficiencies

Our findings suggest two possible mechanisms to explain the pathogenesis of reduced cranial base growth in cleidocranial dysplasia due to *RUNX2* haploinsufficiency in synchondrosis chondrocytes. Firstly, overactive FGFR3-pERK1/2-SOX9 signaling causes elevated COLX expression and premature chondrocyte hypertrophy. Simultaneously, hypertrophic chondrocytes secrete RANKL, which stimulates osteoclasts to degrade the synchondroses. Alternatively, in the absence of *Runx2*, terminally differentiated hypertrophic chondrocytes fail to transdifferentiate into osteoblasts, thus increasing the pool of available hypertrophic cells to produce *Rankl* and stimulate osteoclastogenesis. In both scenarios, local osteoclast-mediated remodeling removes cartilage to allow invasion by osteoblasts with a different origin from those derived from Fgfr3^+^ chondrocytes leading to new bone formation, localized angiogenesis, and synchondrosis fusion. Additionally, increased FGFR3 causes decreased chondrocyte proliferation associated with elevated apoptosis (Fig. 9a, b). It is crucial to acknowledge that while our findings provide valuable insights, they may not encompass all possible etiologies for the phenotypes observed in Fgfr3-Runx2^cKO^ synchondroses. This underscores the importance of delving deeper into the developmental origins of this unique endochondral tissue. Collectively, these studies shed light on the fundamental role of the synchondroses in directing cranial base development and growth and highlight RUNX2 as a key regulator in orchestrating these intricate processes.

## Materials and methods

### Mice

*Fgfr3-iCreER*^*T2*^ (JAX025809), *Rosa26*^*CAG-loxP-stop-loxP-tdTomato*^ (Ai14: R26R-tdTomato, JAX007914), *Col1a1(2.3* *kb)-GFP* (JAX013134) and C57BL/6J (JAX000664) mice were acquired from the Jackson Laboratory. *Runx2*-floxed mice^85^ were provided by Dr. Cecilia Giachelli at the University of Washington. Primers used for *Runx2*-floxed mice include: forward: 5′-CTCAACTTCTCACATGGGTGCT-3′ and reverse: 5′-CTACGGCGGTTGTTTCAATAAGG-3′ with expected sizes of: 851 bp (floxed) and 778 bp (wild type). All procedures were conducted in compliance with the Guidelines for the Care and Use of Laboratory Animals approved by the University of Michigan’s Institutional Animal Care and Use Committee (IACUC), protocols 9496, 9305, and 10975, and the University of Texas Health Science Center at Houston’s Animal Welfare Committee (AWC), protocol AWC-21-0070. All mice were housed in a specific pathogen-free condition and analyzed in a mixed background. Mice were housed in static microisolator cages (Allentown Caging, Allentown, NJ), with ad libitum access to water and food (irradiated LabDiet 5008, Richmond, IN). Animal rooms were climate-controlled, providing temperatures of 22–23 °C and 40%–65% humidity on a 12 h light/dark cycle (lights on at 0600). Both male and female mice were used for the study, as we did not observe any sex bias. *CreER* transgenes were maintained in male breeders for all breeding experiments to avoid spontaneous germline recombination. Mice were identified by micro-tattooing or ear tags. Tail biopsies of mice were lysed by a HotShot protocol (incubating the tail sample at 95 °C for 30 min in an alkaline lysis reagent followed by neutralization) and used for PCR-based genotyping (GoTaq Green Master Mix, Promega, and Nexus X2, Eppendorf). Perinatal mice were also genotyped fluorescently (BLS miner’s lamp) whenever possible. Mice were euthanized by an overdose of carbon dioxide or decapitation under inhalation anesthesia in a drop jar (Fluriso, Isoflurane USP, VetOne, Boise, ID).

### Tamoxifen

Tamoxifen (Sigma-Aldrich, catalog no. T5648) was mixed with 100% ethanol until completely dissolved. Subsequently, a proper volume of sunflower seed oil (Sigma, catalog no. S5007) was added to the tamoxifen-ethanol mixture and rigorously mixed. The tamoxifen-ethanol-oil mixture was incubated at 60 °C in a chemical hood until the ethanol evaporated completely. The tamoxifen-oil mixture was stored at room temperature until use. Tamoxifen (250 μg) was injected intraperitoneally into mice at P3 using a 30-1/2-gauge needle (BD, catalog no. 309597, Franklin Lakes, NJ). Tamoxifen was injected once at a dose of 250 µg at P3.

### Histology and immunofluorescence

Samples were fixed in 4% paraformaldehyde overnight at 4 °C, then decalcified in 15% EDTA (lineage-tracing) or Morse’s solution (immunofluorescence/fluorescent RNAScope) for 1–14 days as appropriate for the tissue size and mouse age. Decalcified samples were cryoprotected in 30% sucrose/PBS solutions and then in 30% sucrose/PBS:OCT (1:1) solutions, each at least overnight at 4 °C. Samples were embedded in an OCT compound (Tissue-Tek, Sakura, Torrance, CA) in the sagittal plane and cryosectioned at 14 µm using a cryostat (Leica CM1850) and adhered to positively charged ColorFrost Plus glass slides (Fisher, Pittsburgh, PA). Sections were postfixed in 4% paraformaldehyde for 15 min at room temperature and then stained with hematoxylin and eosin, Safranin-O/Fast Green, colorimetric Tartrate-resistant acid phosphatase (TRAP) or fluorescent TRAP (ELF97, Invitrogen E6601),^86^ using routine protocol or as previously published. For immunostaining, sections were permeabilized with 0.25% TritonX100/TBS for 30 min, blocked with 3% bovine serum albumin (BSA)/TBS-Tritton X (TBST) for 30 min, and incubated with rabbit anti-Cleaved-Caspase 3 monoclonal antibody (1:100, Cell Signaling, 9664S, Danvers, MA), rabbit anti-COLX polyclonal antibody (1:100, Abcam, ab58632, Cambridge, UK), rabbit anti-FGFR3 polyclonal antibody (1:200, Santa Cruz, sc-123, Santa Cruz, CA), rabbit anti-phospho-ERK1/2 polyclonal antibody (1:100, Cell Signaling, 9102), rabbit anti-Sox9 monoclonal antibody (1:200, Abcam, ab185966), rabbit anti-RUNX2-S319-P antibody (1:500),^41^ mouse anti-CD31 monoclonal antibody (1:100, BD, 553370, Franklin Lakes, NJ) and mouse anti-FLK1 (VEGFR) monoclonal antibody (1:100, BD, 560680) overnight at 4°C, and subsequently with Alexa Fluor 647-conjugated donkey anti-rabbit IgG (1:500, Invitrogen, catalog no. A31573) for 2 h at room temperature. Sections were further incubated with DAPI (4′,6-diamidino-2-phenylindole, 5 µg/mL, Invitrogen D1306, Carlsbad, CA) to stain nuclei prior to imaging.

### Fluorescent RNAscope in situ hybridization

Samples were fixed in 4% paraformaldehyde overnight at 4 °C, decalcified overnight with Morse’s solution, and then cryoprotected. Frozen sections at 12 µm were prepared on positively charged glass slides. In situ hybridization was performed with RNAscope Multiplex Fluorescent Detection Kit v2 (Advanced Cell Diagnostics-Bio-Techne [ACDBio], 323100, Minneapolis, MN) using *Fgfr3* (ACDBio, 440771) and *Tnfrsf11a* (ACDBio, 469581) probes according to the manufacturer’s protocol.

### Imaging

Images were captured using the Leica THUNDER imaging system (Leica Microsystem, Wetzlar, Germany) or by an automated inverted fluorescence microscope with imaged structured illumination system [Zeiss Axio Observer Z1 with ApoTome.2 (Zeiss, Oberkochen, Germany) and Zen 2 (blue edition) software]. For the Zeiss capture system, the filter settings used were: FL Filter Set 34 (Ex. 390/22, Em. 460/50 nm), Set 38 HE (Ex. 470/40, Em. 525/50 nm), Set 43 HE (Ex. 550/25, Em. 605/70 nm), Set 50 (Ex. 640/30, Em. 690/50 nm), and Set 63 HE (Ex. 572/25, Em. 629/62 nm). The objectives used were: Fluar 2.5×/0.12, EC Plan-Neofluar 5×/0.16, Plan-Apochromat 10×/0.45, EC Plan-Neofluar 20×/0.50, EC and Plan-Neofluar 40×/0.75. Images were typically tile-scanned with a motorized stage and reconstructed by a maximum intensity projection (MIP) function. Differential interference contrast (DIC) was used for objectives higher than 10x. Representative images of at least three independent biological samples are shown in the figures. Quantification of cells and fluorescence intensity on sections was performed blindly using NIH Image J or via a machine learning approach using the AIVIA image analysis software (Leica Microsystem) on primary spongiosa region and chondrocyte zones. The DIC channel was removed for quantification to allow single-channel analysis.

### Three-dimensional microcomputed tomography analysis

Specimens were placed in a 19-mm diameter specimen holder and scanned using a micro-CT system (µCT100 Scanco Medical, Bassersdorf, Switzerland). Scan settings were: voxel size 12 µm, 70 kVp, 114 µA, 0.5-mm AL filter, and integration time 500 ms. Linear image analysis was performed using Dragonfly image analysis software (Comet, Montréal, Québec) on 2D images in the x-y, y-z, and x-z planes. 3D-rendered skulls were generated using Dragonfly.

### EdU

5-Ethynyl-2′-deoxyuridine (EdU) (Invitrogen, catalog no. A10044) dissolved in PBS was administered to mice at indicated postnatal days. Click-iT Imaging Kit with Alexa Flour 647-azide (Invitrogen, catalog no. C10640) was used to detect EdU in cryosections. Fgfr3-CE^tdT^ mice received one dose of EdU (50 µg each) 3 h prior to euthanasia.

### Replicates

All experiments were performed in biological replicates. To ensure reproducibility, we examined at least three independent biological samples (three different mice) for all data presented in the manuscript. Biological replicates were defined as multiple experimental samples sharing common genotypes and genetic backgrounds. For each series of experiments, all attempts at biological replication were successful. Technical replicates were generated from a single experimental sample. For example, serial sections of the synchondroses from a single mouse were considered technical replicates. All quantitative data were included to ensure transparency in our data interpretation.

### Statistical analysis

Graphs display mean ± standard deviation (s.d.), and each data point represents an independent mouse. Statistical evaluation was conducted based on Mann-Whitney’s *U*-test using GraphPad Prism. A *P*-value < 0.05 was considered statistically significant. No statistical method was used to predetermine the sample size. The sample size was determined based on previous literature and our previous experience to give sufficient standard deviations of the mean so as not to miss a biologically important difference between groups. The experiments were not randomized. All the available mice with the desired genotypes were used for experiments.

## Supplementary information

Supplementary Figures

Supplementary Figure Legends

## Data Availability

All data needed to evaluate the conclusions in the paper are present in the paper and/or the Supplementary Materials.

## References

[1] Venugopalan, S. R. & Van Otterloo, E. The skull’s girder: a brief review of the cranial base. *J. Dev. Biol.* **9**, 3 (2021). 33498686 10.3390/jdb9010003 PMC7838769

[2] Cendekiawan, T., Wong, R. W. K. & Rabie, A. B. M. Relationships between cranial base synchondroses and craniofacial development: a review. *Open Anat. J.* **2**, 67–75 (2010).

[3] Petrovic, A. & Charlier, J. P. The spheno-occipital synchondrosis of the young rat in organ culture: demonstration of a potential of independent growth. *C. R. Acad. Hebd. Seances Acad. Sci. D.* **265**, 1511–1513 (1967). 4967605

[4] Servoss, J. M. An in vivo and in vitro autoradiographic investigation of growth in synchondrosal cartilage. *Am. J. Anat.* **136**, 479–485 (1973). 4692974 10.1002/aja.1001360407

[5] Kuroda, T., Miura, F., Nakamura, T. & Noguchi, K. Cellular kinetics of synchondrosal cartilage in organ culture. *Proc. Finn. Dent. Soc.* **77**, 89 (1981). 7255413

[6] Alhazmi, A. et al. Timing and rate of spheno-occipital synchondrosis closure and its relationship to puberty. *PLoS ONE* **12**, e0183305 (2017). Erratum in: *PLoS One*. **13**(1), e0191703 (2018). 29352313 10.1371/journal.pone.0191703 PMC5774834

[7] Madeline, L. A. & Elster, A. D. Postnatal development of the central skull base: normal variants. *Radiology* **196**, 757–763 (1995). 7644640 10.1148/radiology.196.3.7644640

[8] Vu, G. et al. Physiologic timeline of cranial-base suture and synchondrosis closure. *Plast. Reconstr. Surg.* **148**, 973e–982e (2021). 34705810 10.1097/PRS.0000000000008570

[9] Scott, J. H. The cranial base. *Am. J. Phys. Anthropol.* **16**, 319–348 (1958). 13649900 10.1002/ajpa.1330160305

[10] Wei, X., Thomas, N., Hatch, N. E., Hu, M. & Liu, F. Postnatal craniofacial skeletal development of female C57BL/6NCrl mice. *Front. Phys.* **8**, 697 (2017). 10.3389/fphys.2017.00697 PMC5603710 28959213

[11] Rijken, B. M., Lequin, M. H., de Rooi, J. J., van Veelen, M. C. & Mathijssen, I. J. Foramen magnum size and involvement of its intraoccipital synchondroses in Crouzon Syndrome. *Plast. Reconstr. Surg.* **132**, 993e–1000e (2013). 24281646 10.1097/PRS.0b013e3182a8077e

[12] Cheung, M. S. et al. Achondroplasia foramen magnum score: screening infants for stenosis. *Arch. Dis. Child.* **106**, 180–184 (2021). 32883660 10.1136/archdischild-2020-319625

[13] Hallett, S. A., Ono, W. & Ono, N. Growth plate chondrocytes: skeletal development, growth and beyond. *Int. J. Mol. Sci.* **20**, 6009 (2019). 31795305 10.3390/ijms20236009 PMC6929081

[14] Kronenberg, H. M. Developmental regulation of the growth plate. *Nature* **423**, 332–336 (2003). 12748651 10.1038/nature01657

[15] Hallett, S. A., Ono, W., Franceschi, R. T. & Ono, N. Cranial base synchondrosis: chondrocytes at the hub. *Int. J. Mol. Sci.* **23**, 7817 (2022). 35887171 10.3390/ijms23147817 PMC9317907

[16] Wei, X., Hu, M., Mishina, Y. & Liu, F. Developmental regulation of the growth plate and cranial synchondrosis. *J. Dent. Res.* **95**, 1221–1229 (2016). 27250655 10.1177/0022034516651823 PMC5076755

[17] Funato, N. New insights Into cranial synchondrosis development: a mini review. *Front. Cell Dev. Biol.* **8**, 1–9 (2020). 32850826 10.3389/fcell.2020.00706 PMC7432265

[18] Twigg, S. R. F. & Wilkie, A. O. M. New insights into craniofacial malformations. *Hum. Mol. Gen.* **24**, R50–R59 (2015). 26085576 10.1093/hmg/ddv228 PMC4571997

[19] Yang, J. H. et al. Time and pattern of the fusion of the spheno-occipital synchondrosis in patients with skeletal Class I and Class III malocclusion. *Angle Orthod.* **8**, 470–479 (2019). 10.2319/052218-386.1 PMC8117700 30516418

[20] Tahiri, Y., Paliga, J. T., Vossough, A., Bartlett, S. P. & Taylor, J. A. The spheno-occipital synchondrosis fuses prematurely in patients with Crouzon syndrome and midface hypoplasia compared with age- and gender-matched controls. *J. Oral. Maxillofac. Surg.* **72**, 1173–1179 (2014). 24480760 10.1016/j.joms.2013.11.015

[21] Paliga, J. T., Goldstein, J. A., Vossough, A., Bartlett, S. P. & Taylor, J. A. Premature closure of the spheno-occipital synchondrosis in Pfeiffer syndrome: a link to midface hypoplasia. *J. Craniofacial Surg.* **25**, 202–205 (2014). 10.1097/SCS.0000000000000386 24406578

[22] Allareddy, V. et al. Craniofacial features as assessed by lateral cephalometric measurements in children with Down syndrome. *Prog. Orthod.* **17**, 35 (2016). 27722998 10.1186/s40510-016-0148-7 PMC5097953

[23] Brkic, H., Kaic, Z., Poje, Z. & Singer, Z. Shape of the craniofacial complex in patients with Klinefelter syndrome. *Angle Orthod.* **64**, 371–376 (1994). 7802331 10.1043/0003-3219(1994)064<0371:SOTCCI>2.0.CO;2

[24] Pauli, R. M. Achondroplasia: a comprehensive clinical review. *Orphanet J. Rare Dis.* **14**, 1 (2019). 30606190 10.1186/s13023-018-0972-6 PMC6318916

[25] Combs, P. D. & Harshbarger, R. J. Le Fort I maxillary advancement using distraction osteogenesis. *Semin. Plast. Surg.* **28**, 193–198 (2014). 25383054 10.1055/s-0034-1390172 PMC4219915

[26] Andria, L. M., Leite, L. P., Prevatte, T. M. & King, L. B. Correlation of the cranial base angle and its components with other dental/skeletal variables and treatment time. *Angle Orthod.* **74**, 361–366 (2004). 15264648 10.1043/0003-3219(2004)074<0361:COTCBA>2.0.CO;2

[27] Komori, T. et al. Targeted disruption of Cbfa1 results in a complete lack of bone formation owing to maturational arrest of osteoblasts. *Cell* **89**, 755–764 (1997). 9182763 10.1016/s0092-8674(00)80258-5

[28] Komori, T. Molecular mechanism of runx2-dependent bone development. *Mol. Cells* **43**, 168–175 (2020). 31896233 10.14348/molcells.2019.0244 PMC7057844

[29] Pan, C. Y., Tseng, Y. C., Lan, T. H. & Chang, H. P. Craniofacial features of cleidocranial dysplasia. *J. Dent. Sci.* **12**, 313–318 (2017). 30895069 10.1016/j.jds.2017.07.002 PMC6395362

[30] Jensen, B. L. & Kreiborg, S. Craniofacial growth in cleidocranial dysplasia-a roentgencephalometric study. *J. Cranio. Genet. Dev. Biol.* **15**, 35–43 (1995). 7601912

[31] Al Kaissi, A. et al. Broad spectrum of skeletal malformation complex in patients with cleidocranial dysplasia syndrome: radiographic and tomographic study. *Clin. Med. Insights Arthritis Musculoskelet. Disord.* **19**, 45–55 (2013). 10.4137/CMAMD.S11933 PMC3762605 24023524

[32] Jin, R. et al. Inhibition of miR338 rescues cleidocranial dysplasia in Runx2 mutant mice partially via the Hif1a-Vegfa axis. *Exp. Mol. Med.* **55**, 69–80 (2023). 36599929 10.1038/s12276-022-00914-w PMC9898552

[33] Shiang, R. et al. Mutations in the transmembrane domain of FGFR3 cause the most common genetic form of dwarfism, achondroplasia. *Cell* **78**, 335–342 (1994). 7913883 10.1016/0092-8674(94)90302-6

[34] Bonaventure, J. et al. Mutations in the gene encoding fibroblast growth factor receptor 3 account for achondroplasia, hypochondroplasia and thanatophoric dysplasia. *Acta Paediatr. Suppl.* **417**, 33–38 (1996). 9055906 10.1111/j.1651-2227.1996.tb14291.x

[35] Matsushita, T. et al. FGFR3 promotes synchondrosis closure and fusion of ossification centers through the MAPK pathway. *Hum. Mol. Genet.* **18**, 227–240 (2009). 18923003 10.1093/hmg/ddn339 PMC2638772

[36] Murakami, S. et al. Constitutive activation of MEK1 in chondrocytes causes Stat1-independent achondroplasia-like dwarfism and rescues the Fgfr3-deficient mouse phenotype. *Genes Dev.* **18**, 290–305 (2004). 14871928 10.1101/gad.1179104 PMC338282

[37] Kawane, T. et al. Runx2 is required for the proliferation of osteoblast progenitors and induces proliferation by regulating Fgfr2 and Fgfr3. *Sci. Rep.* **8**, 13551 (2018). 30202094 10.1038/s41598-018-31853-0 PMC6131145

[38] Rivers, L. E. et al. PDGFRA/NG2 glia generate myelinating oligodendrocytes and piriform projection neurons in adult mice. *Nat. Neurosci.* **11**, 1392–1401 (2008). 18849983 10.1038/nn.2220 PMC3842596

[39] Matsushita, Y. et al. The fate of early perichondrial cells in developing bones. *Nat. Commun.* **13**, 7319 (2022). 36443296 10.1038/s41467-022-34804-6 PMC9705540

[40] Matsushita, Y. et al. Bone marrow endosteal stem cells dictate active osteogenesis and aggressive tumorigenesis. *Nat. Commun.* **14**, 23 83 (2023). 37185464 10.1038/s41467-023-38034-2 PMC10130060

[41] Vora, S. R., Camci, E. D. & Cox, T. C. Postnatal ontogeny of the cranial base and craniofacial skeleton in male C57BL/6J mice: a reference standard for quantitative analysis. *Front. Physiol.* **6**, 417 (2016). 26793119 10.3389/fphys.2015.00417 PMC4709510

[42] Ge, C. et al. Interactions between extracellular signal-regulated kinase 1/2 and p38 MAP kinase pathways in the control of RUNX2 phosphorylation and transcriptional activity. *J. Bone Miner. Res.* **27**, 538–551 (2012). Erratum in: *J. Bone. Miner. Res*. **36**(10), 2096-2097 (2021). 34004069 10.1002/jbmr.4300

[43] Thomas, C. M., Fuller, C. J., Whittles, C. E. & Sharif, M. Chondrocyte death by apoptosis is associated with cartilage matrix degradation. *Osteoarthr. Cartil.* **15**, 27–34 (2007). 10.1016/j.joca.2006.06.012 16859932

[44] Kartsogiannis, V. et al. Localization of RANKL (receptor activator of NF kappa B ligand) mRNA and protein in skeletal and extraskeletal tissues. *Bone* **25**, 525–534 (1999). 10574572 10.1016/s8756-3282(99)00214-8

[45] Gerber, H. P. et al. VEGF couples hypertrophic cartilage remodeling, ossification and angiogenesis during endochondral bone formation. *Nat. Med.* **5**, 623–628 (1999). 10371499 10.1038/9467

[46] Lertkiatmongkol, P., Liao, D., Mei, H., Hu, Y. & Newman, P. J. Endothelial functions of platelet/endothelial cell adhesion molecule-1 (CD31). *Curr. Opin. Hematol.* **23**, 253–259 (2016). 27055047 10.1097/MOH.0000000000000239 PMC4986701

[47] Shibuya, M. Vascular endothelial growth factor (VEGF) and its receptor (VEGFR) signaling in angiogenesis: a crucial target for anti- and pro-angiogenic therapies. *Genes Cancer* **2**, 1097–1105 (2011). 22866201 10.1177/1947601911423031 PMC3411125

[48] Raucci, A., Laplantine, E., Mansukhani, A. & Basilico, C. Activation of the ERK1/2 and p38 mitogen-activated protein kinase pathways mediates fibroblast growth factor-induced growth arrest of chondrocytes. *J. Biol. Chem.* **279**, 1747–1756 (2004). 14593093 10.1074/jbc.M310384200

[49] Murakami, S., Kan, M., McKeehan, W. L. & de Crombrugghe, B. Up-regulation of the chondrogenic Sox9 gene by fibroblast growth factors is mediated by the mitogen-activated protein kinase pathway. *Proc. Natl. Acad. Sci. USA* **97**, 1113–1118 (2000). 10655493 10.1073/pnas.97.3.1113 PMC15539

[50] Komori, T. Regulation of proliferation, differentiation and functions of osteoblasts by Runx2. *Int. J. Mol. Sci.* **20**, 1694 (2019). 30987410 10.3390/ijms20071694 PMC6480215

[51] Maeno, T. et al. Early onset of Runx2 expression caused craniosynostosis, ectopic bone formation, and limb defects. *Bone* **49**, 673–682 (2011). 21807129 10.1016/j.bone.2011.07.023

[52] Presto, P., Collins, R. A., Garza, J., Zeitouni, O. F. & Nagy, L. Sex differences in comorbidities of pediatric craniosynostosis at presentation. *Pediatr. Neurosurg.* **58**, 8–17 (2023). 36543149 10.1159/000528745 PMC10064380

[53] Chen, H. et al. Runx2 regulates endochondral ossification through control of chondrocyte proliferation and differentiation. *J. Bone Miner. Res.* **29**, 2653–2665 (2014). 24862038 10.1002/jbmr.2287 PMC4535340

[54] Takarada, T. et al. An analysis of skeletal development in osteoblast-specific and chondrocyte-specific runt-related transcription factor-2 (Runx2) knockout mice. *J. Bone Miner. Res.* **28**, 2064–2069 (2013). 23553905 10.1002/jbmr.1945

[55] Qin, X. et al. Runx2 is essential for the transdifferentiation of chondrocytes into osteoblasts. *PLoS Genet.* **16**, e1009169 (2020). 33253203 10.1371/journal.pgen.1009169 PMC7728394

[56] Chen, H. et al. Chondrocyte-specific regulatory activity of Runx2 is essential for survival and skeletal development. *Cells Tiss. Organs* **194**, 161–165 (2011). 10.1159/000324743 PMC3178074 21597273

[57] Rashid, H., Smith, C. M., Convers, V., Clark, K. & Javed, A. Runx2 deletion in hypertrophic chondrocytes impairs osteoclast mediated bone resorption. *Bone* **181**, 117014 (2024). 38218304 10.1016/j.bone.2024.117014 PMC10922707

[58] Cooper, K. L. et al. Multiple phases of chondrocyte enlargement underlie differences in skeletal proportions. *Nature* **495**, 375–378 (2013). 23485973 10.1038/nature11940 PMC3606657

[59] Hallett, S. A., Ono, W. & Ono, N. The hypertrophic chondrocyte: to be or not to be. *Histol. Histopathol.* **36**, 1021–1036 (2021). 34137454 10.14670/HH-18-355 PMC8678381

[60] Linsenmayer, T. F. et al. Collagen types IX and X in the developing chick tibiotarsus: analyses of mRNAs and proteins. *Development* **111**, 191–196 (1991). 2015794 10.1242/dev.111.1.191

[61] Drissi, M. H. et al. Runx2/Cbfa1 stimulation by retinoic acid is potentiated by BMP2 signaling through interaction with Smad1 on the collagen X promoter in chondrocytes. *J. Cell. Biochem.* **90**, 1287–1298 (2003). 14635200 10.1002/jcb.10677

[62] Zheng, Q. et al. Type X collagen gene regulation by Runx2 contributes directly to its hypertrophic chondrocyte-specific expression in vivo. *J. Cell Biol.* **162**, 833–842 (2003). 12952936 10.1083/jcb.200211089 PMC2172833

[63] Yoshida, C. A. et al. Runx2 and Runx3 are essential for chondrocyte maturation, and Runx2 regulates limb growth through induction of Indian hedgehog. *Genes Dev.* **18**, 952–963 (2004). 15107406 10.1101/gad.1174704 PMC395853

[64] Takeda, S., Bonnamy, J. P., Owen, M. J., Ducy, P. & Karsenty, G. Continuous expression of Cbfa1 in nonhypertrophic chondrocytes uncovers its ability to induce hypertrophic chondrocyte differentiation and partially rescues Cbfa1-deficient mice. *Genes Dev.* **15**, 467–481 (2001). 11230154 10.1101/gad.845101 PMC312629

[65] Enomoto, H. et al. Cbfa1 is a positive regulatory factor in chondrocyte maturation. *J. Biol. Chem.* **275**, 8695–8702 (2000). 10722711 10.1074/jbc.275.12.8695

[66] Kishimoto, K., Kitazawa, R., Kurosaka, M., Maeda, S. & Kitazawa, S. Expression profile of genes related to osteoclastogenesis in mouse growth plate and articular cartilage. *Histochem. Cell Biol.* **125**, 593–602 (2006). 16283360 10.1007/s00418-005-0103-z

[67] Wada, T., Nakashima, T., Hiroshi, N. & Penninger, J. M. RANKL-RANK signaling in osteoclastogenesis and bone disease. *Trends Mol. Med.* **12**, 17–25 (2006). 16356770 10.1016/j.molmed.2005.11.007

[68] Xiong, J. et al. Matrix-embedded cells control osteoclast formation. *Nat. Med.* **17**, 1235–1241 (2011). 21909103 10.1038/nm.2448 PMC3192296

[69] Grosso, A. et al. It takes two to tango: coupling of angiogenesis and osteogenesis for bone regeneration. *Front. Bioeng. Biotechnol.* **5**, 68 (2017). 29164110 10.3389/fbioe.2017.00068 PMC5675838

[70] Wang, Y. et al. A mouse model for achondroplasia produced by targeting fibroblast growth factor receptor 3. *Proc. Natl. Acad. Sci. USA* **96**, 4455–4460 (1999). 10200283 10.1073/pnas.96.8.4455 PMC16353

[71] Su, N. et al. Gain-of-function mutation in FGFR3 in mice leads to decreased bone mass by affecting both osteoblastogenesis and osteoclastogenesis. *Hum. Mol. Genet.* **19**, 1199–1210 (2010). 20053668 10.1093/hmg/ddp590 PMC3115638

[72] Gibbs, L. & Legeai-Mallet, L. FGFR3 intracellular mutations induce tyrosine phosphorylation in the Golgi and defective glycosylation. *Biochim. Biophys. Acta* **1773**, 502–512 (2007). 17320202 10.1016/j.bbamcr.2006.12.010

[73] Zhou, L. et al. Nuclear translocation of fibroblast growth factor receptor 3 and its significance in pancreatic cancer. *Int. J. Clin. Exp. Pathol.* **8**, 14640–14648 (2015). 26823787 PMC4713573

[74] Zammit, C. et al. Altered intracellular localization of fibroblast growth factor receptor 3 in human breast cancer. *J. Pathol.* **194**, 27–34 (2001). 11329138 10.1002/path.846

[75] Ornitz, D. M. & Marie, P. J. Fibroblast growth factor signaling in skeletal development and disease. *Genes Dev.* **29**, 1463–1486 (2015). 26220993 10.1101/gad.266551.115 PMC4526732

[76] Matsushita, T. et al. Extracellular signal-regulated kinase 1 (ERK1) and ERK2 play essential roles in osteoblast differentiation and in supporting osteoclastogenesis. *Mol. Cell. Biol.* **29**, 5843–5857 (2009). 19737917 10.1128/MCB.01549-08 PMC2772724

[77] Matsushita, M. et al. Meclozine facilitates proliferation and differentiation of chondrocytes by attenuating abnormally activated FGFR3 signaling in achondroplasia. *PLoS ONE* **8**, e81569 (2013). 24324705 10.1371/journal.pone.0081569 PMC3852501

[78] Matsushita, M. et al. Meclozine promotes longitudinal skeletal growth in transgenic mice with achondroplasia carrying a gain-of-function mutation in the FGFR3 gene. *Endocrinology* **156**, 548–554 (2015). 25456072 10.1210/en.2014-1914

[79] Bi, W., Deng, J. M., Zhang, Z., Behringer, R. R. & de Crombrugghe, B. Sox9 is required for cartilage formation. *Nat. Genet.* **22**, 85–89 (1999). 10319868 10.1038/8792

[80] Oh, C. D. et al. SOX9 regulates multiple genes in chondrocytes, including genes encoding ECM proteins, ECM modification enzymes, receptors, and transporters. *PLoS ONE* **9**, e107577 (2014). Erratum in*: PLoS One*. **10**(11), e0143156 (2015). 26562508 10.1371/journal.pone.0143156 PMC4642988

[81] Leung, V. Y. et al. SOX9 governs differentiation stage-specific gene expression in growth plate chondrocytes via direct concomitant transactivation and repression. *PLoS Genet.* **7**, e1002356 (2011). 22072985 10.1371/journal.pgen.1002356 PMC3207907

[82] Haseeb, A. et al. SOX9 keeps growth plates and articular cartilage healthy by inhibiting chondrocyte dedifferentiation/osteoblastic redifferentiation. *Proc. Natl. Acad. Sci. USA* **118**, e2019152118 (2021). 33597301 10.1073/pnas.2019152118 PMC7923381

[83] Hattori, T. et al. SOX9 is a major negative regulator of cartilage vascularization, bone marrow formation and endochondral ossification. *Development* **137**, 901–911 (2010). 20179096 10.1242/dev.045203

[84] Lui, J. C. et al. Persistent Sox9 expression in hypertrophic chondrocytes suppresses transdifferentiation into osteoblasts. *Bone* **125**, 169–177 (2019). 31121357 10.1016/j.bone.2019.05.027 PMC7558415

[85] Lin, M. E., Chen, T., Leaf, E. M., Speer, M. Y. & Giachelli, C. M. Runx2 expression in smooth muscle cells Is required for arterial Medial calcification in mice. *Am. J. Pathol.* **185**, 1958–1969 (2015). 25987250 10.1016/j.ajpath.2015.03.020 PMC4484217

[86] Filgueira, L. Fluorescence-based staining for tartrate-resistant acidic phosphatase (TRAP) in osteoclasts combined with other fluorescent dyes and protocols. *J. Histochem. Cytochem.* **52**, 411–414 (2004). 14966208 10.1177/002215540405200312

