# Supplementary material for: RUNX2 is essential for maintaining synchondrosis chondrocytes and cranial base growth
Source: Bone Res. 2025 May 29;13:57. doi: 10.1038/s41413-025-00426-z (PMC12122814; doi:10.1038/s41413-025-00426-z)

Supplemental Figure 1

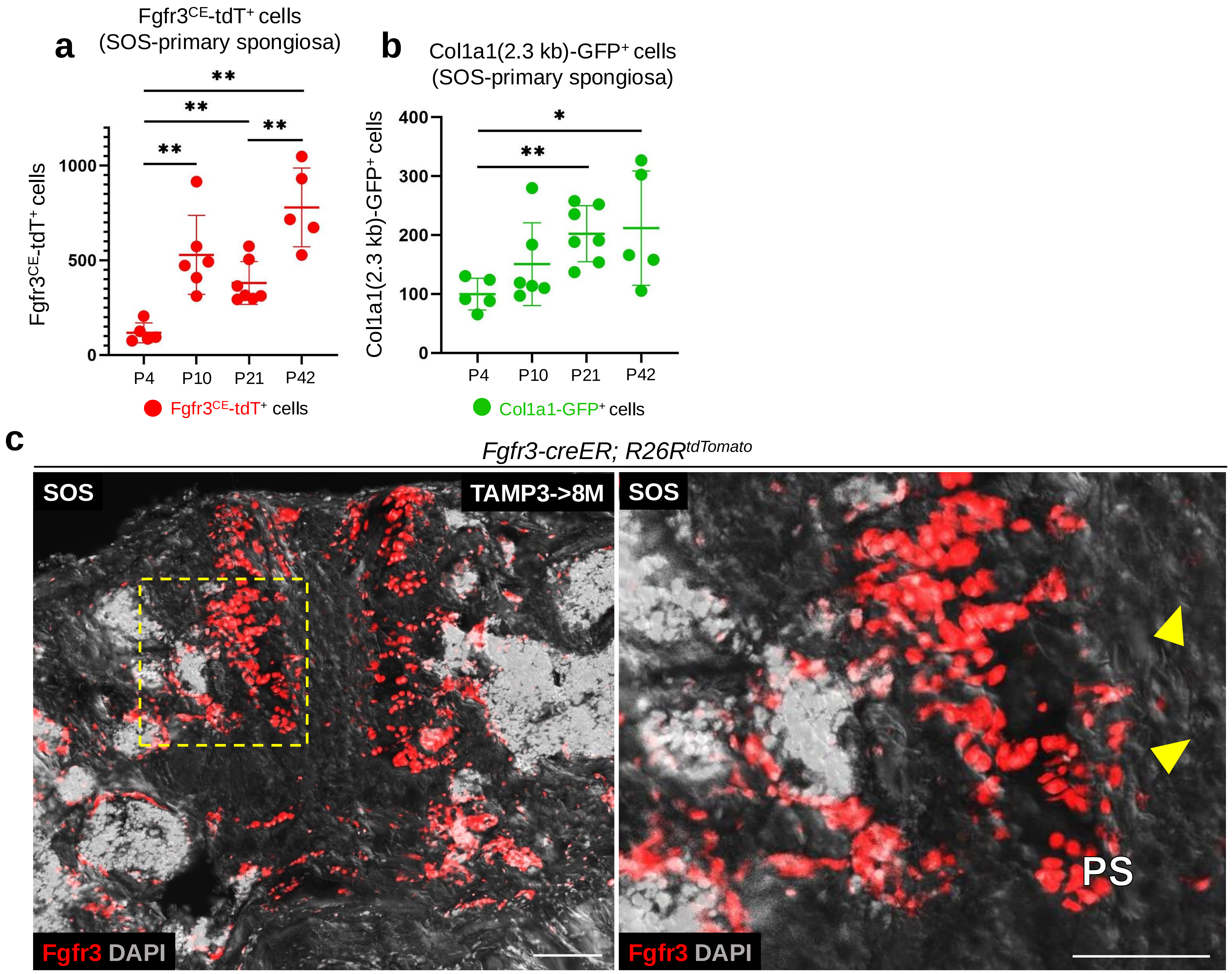

Supplemental Figure 2

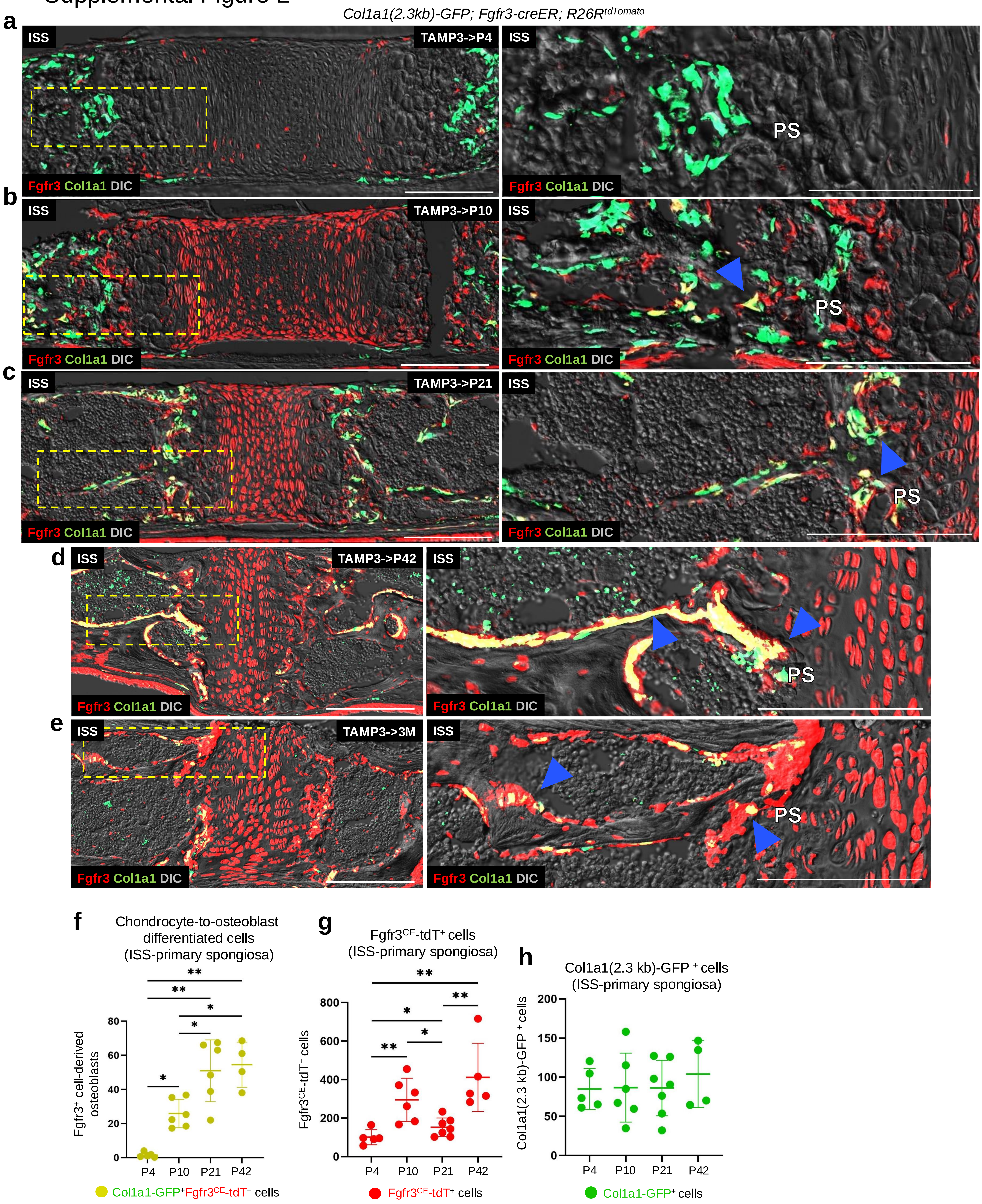

Supplemental Figure 3

a

Col1a1(2.3kb)-GFP; Fgfr3-creER; R26R^{tdTomato}

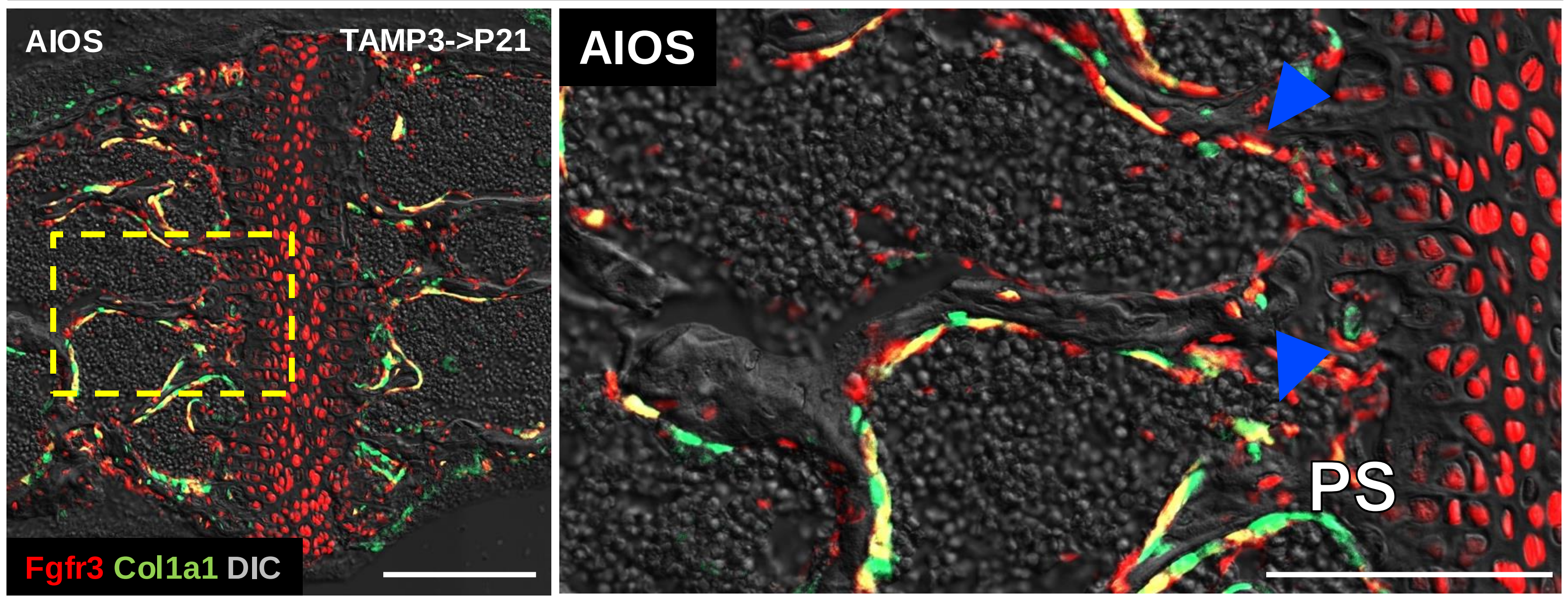

Supplemental Figure 4

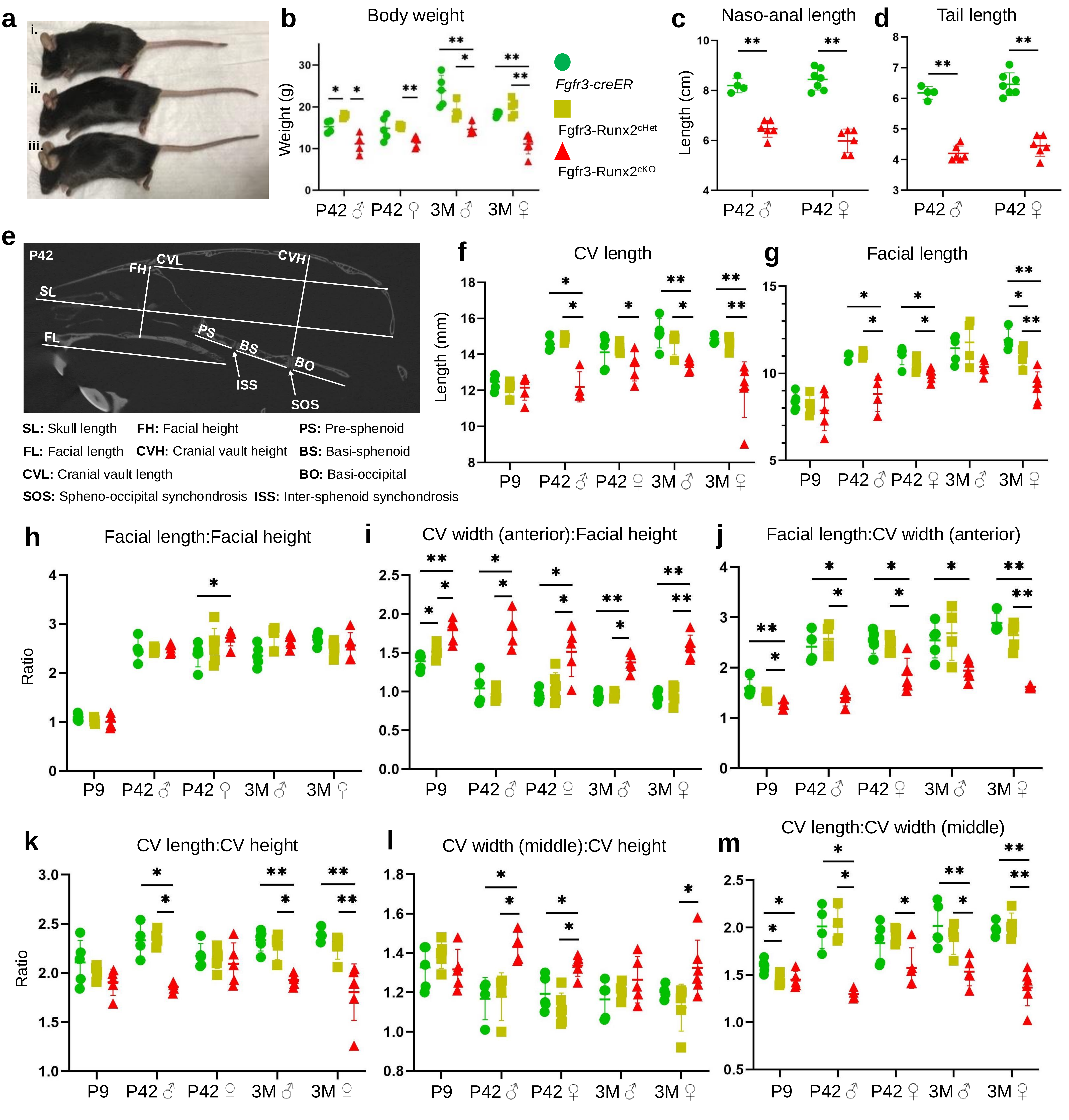

Supplemental Figure 5

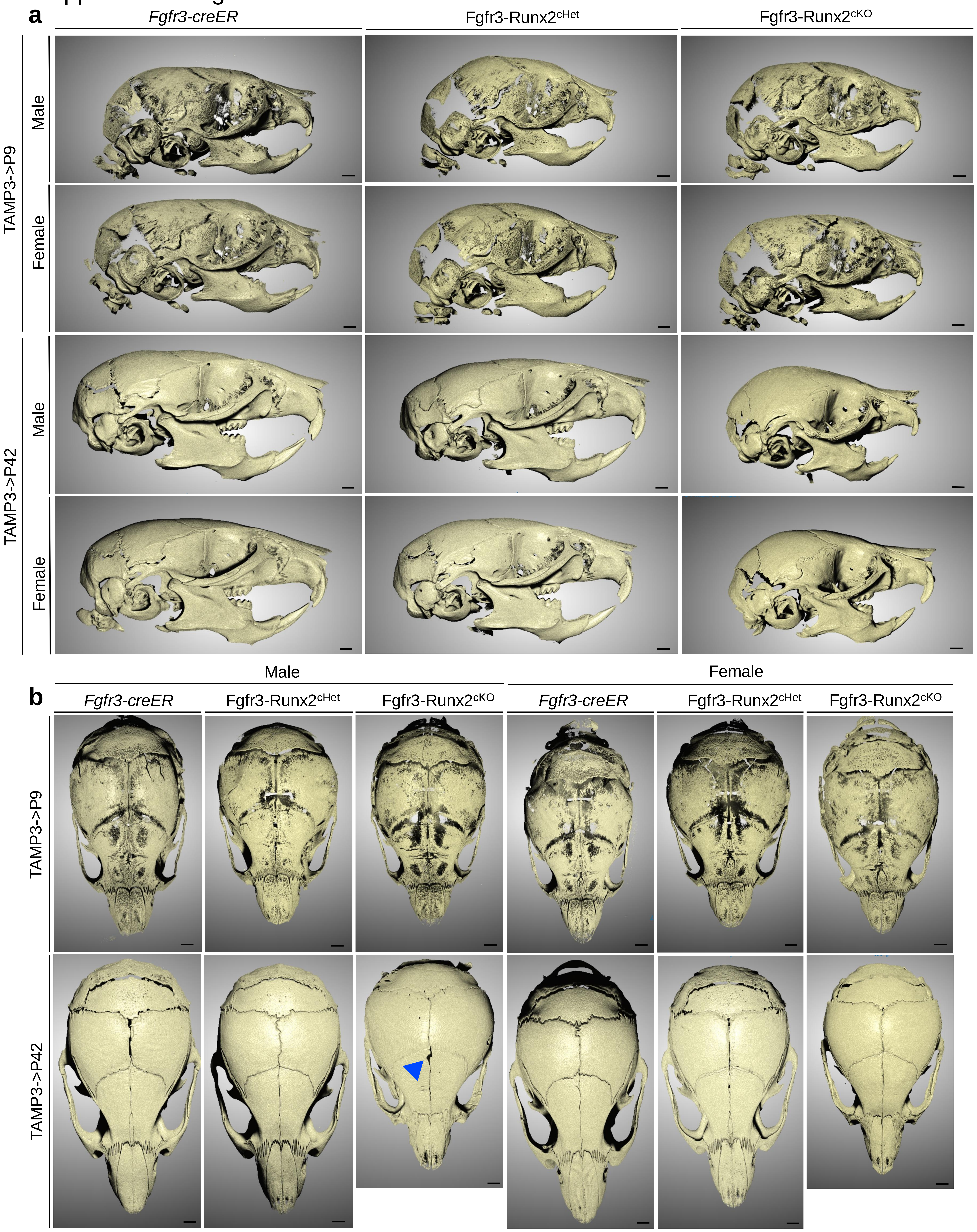

Supplemental Figure 6

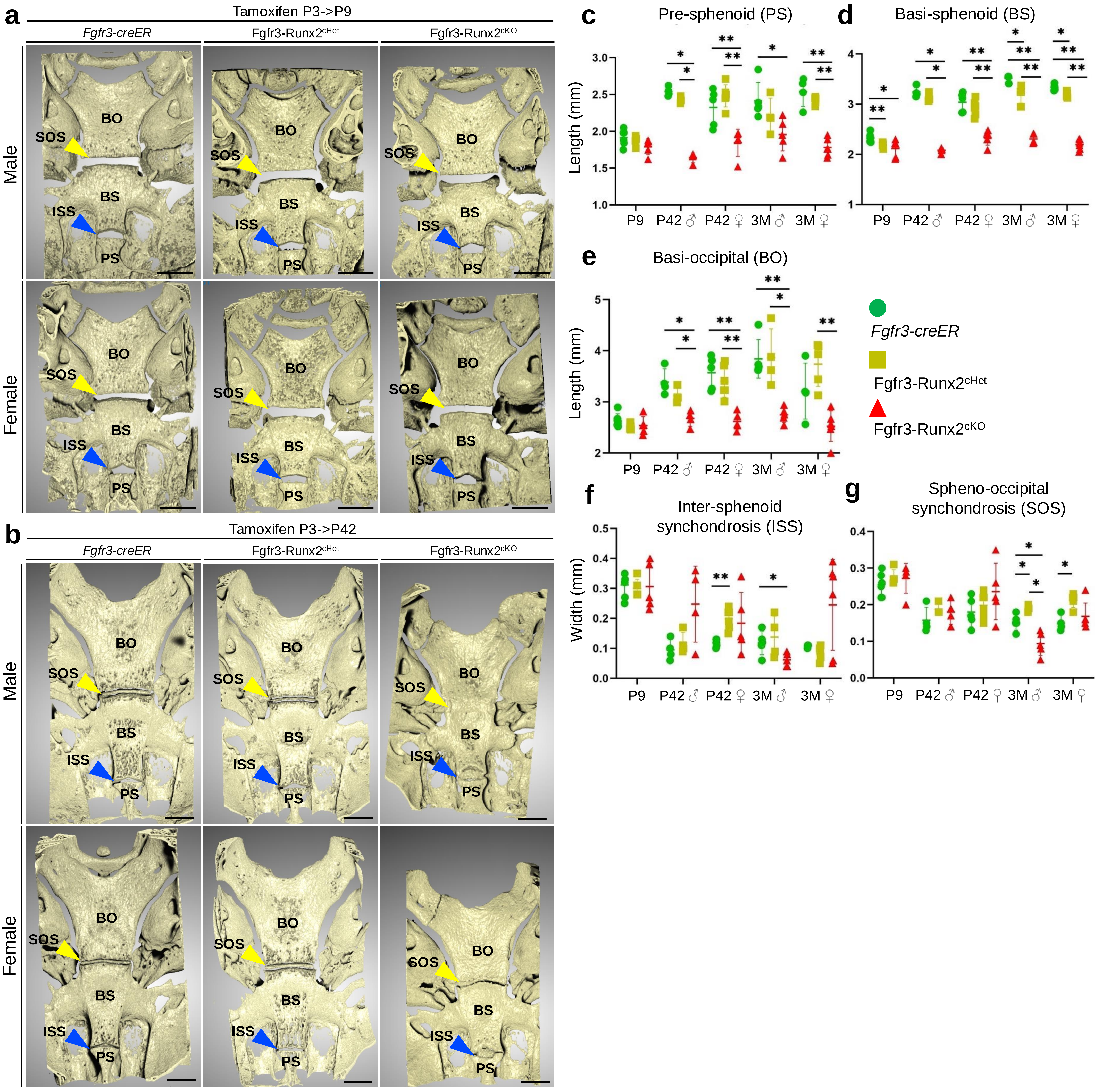

Supplemental Figure 7

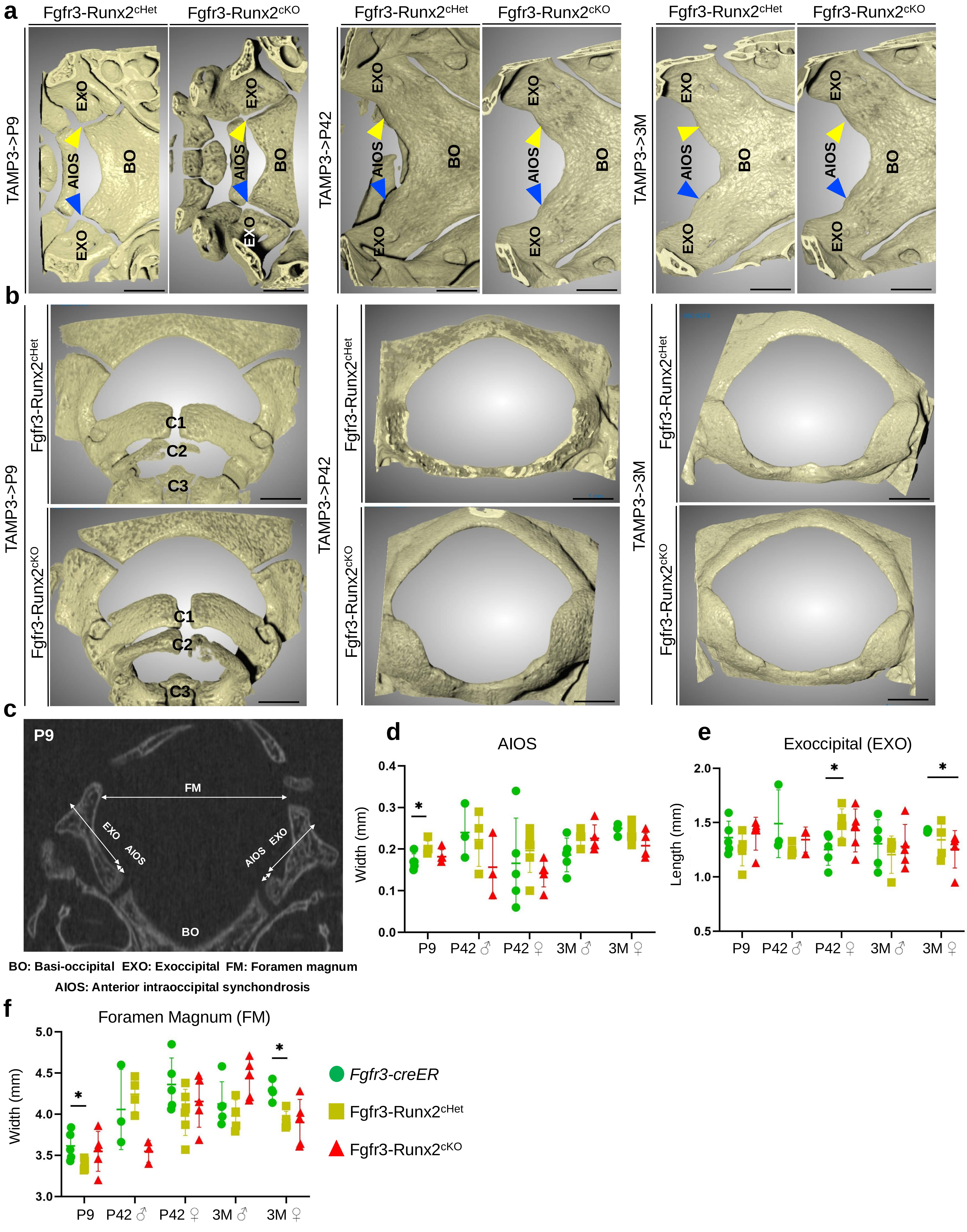

BO: Basi-occipital EXO: Exoccipital FM: Foramen magnum

AIOS: Anterior intraoccipital synchondrosis

Supplemental Figure 8

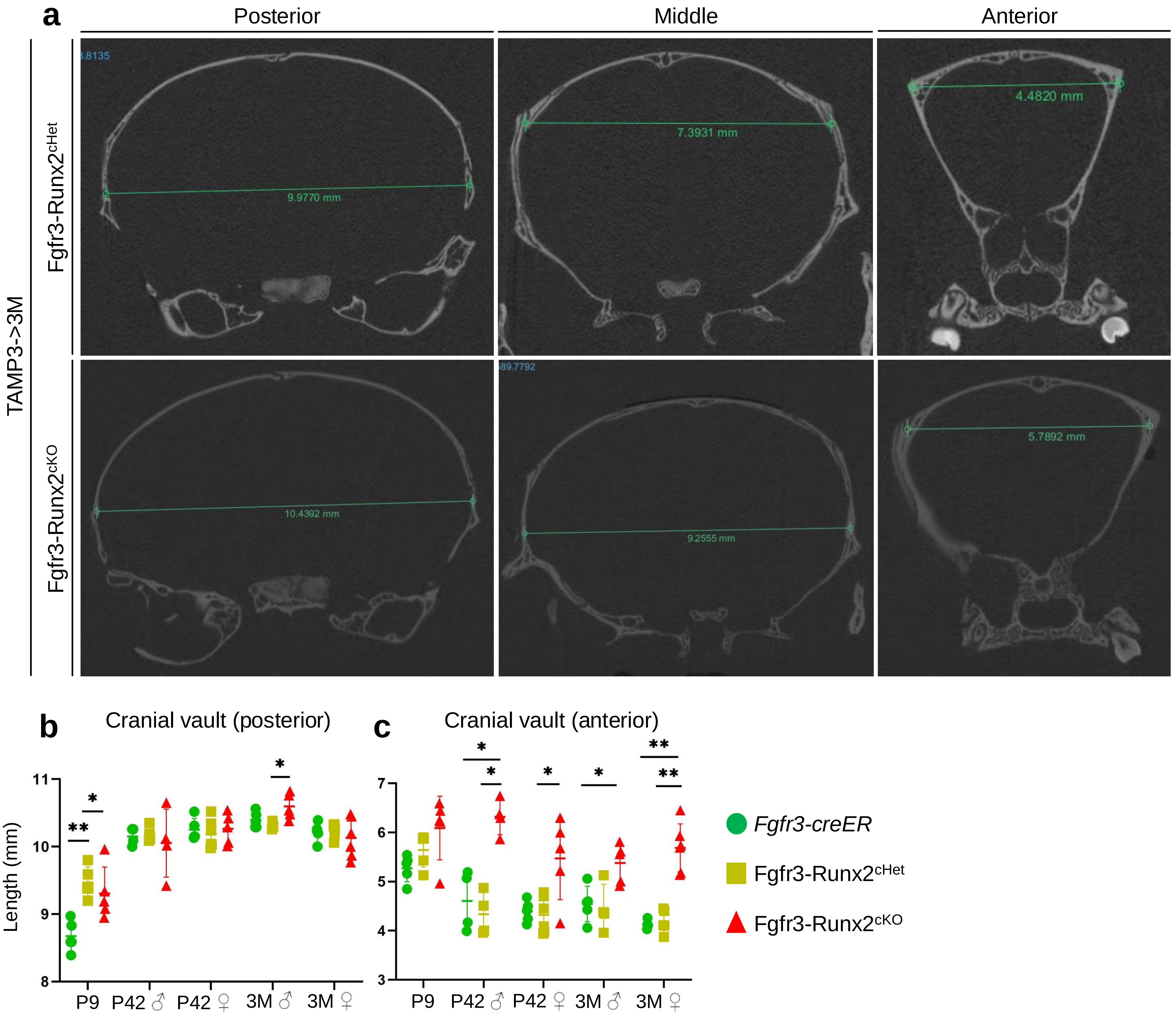

Supplemental Figure 9

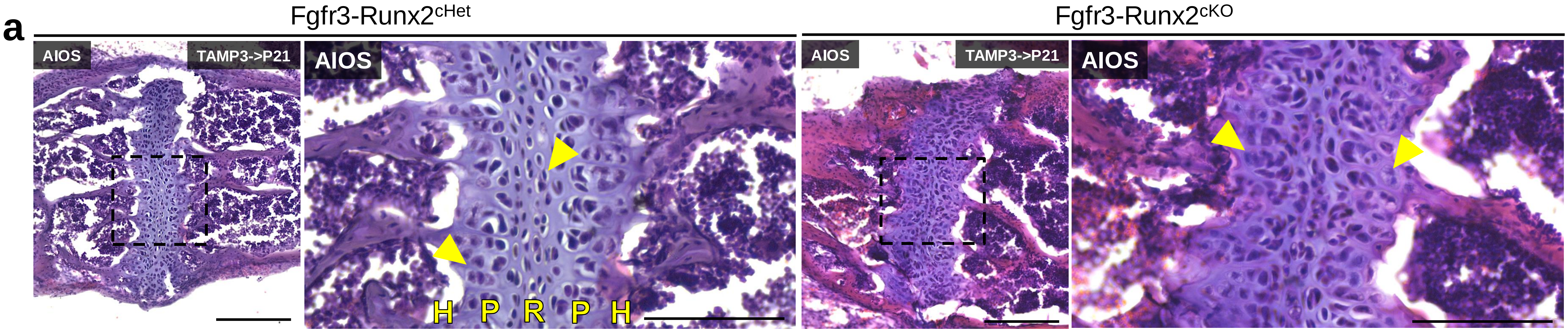

Supplemental Figure 10

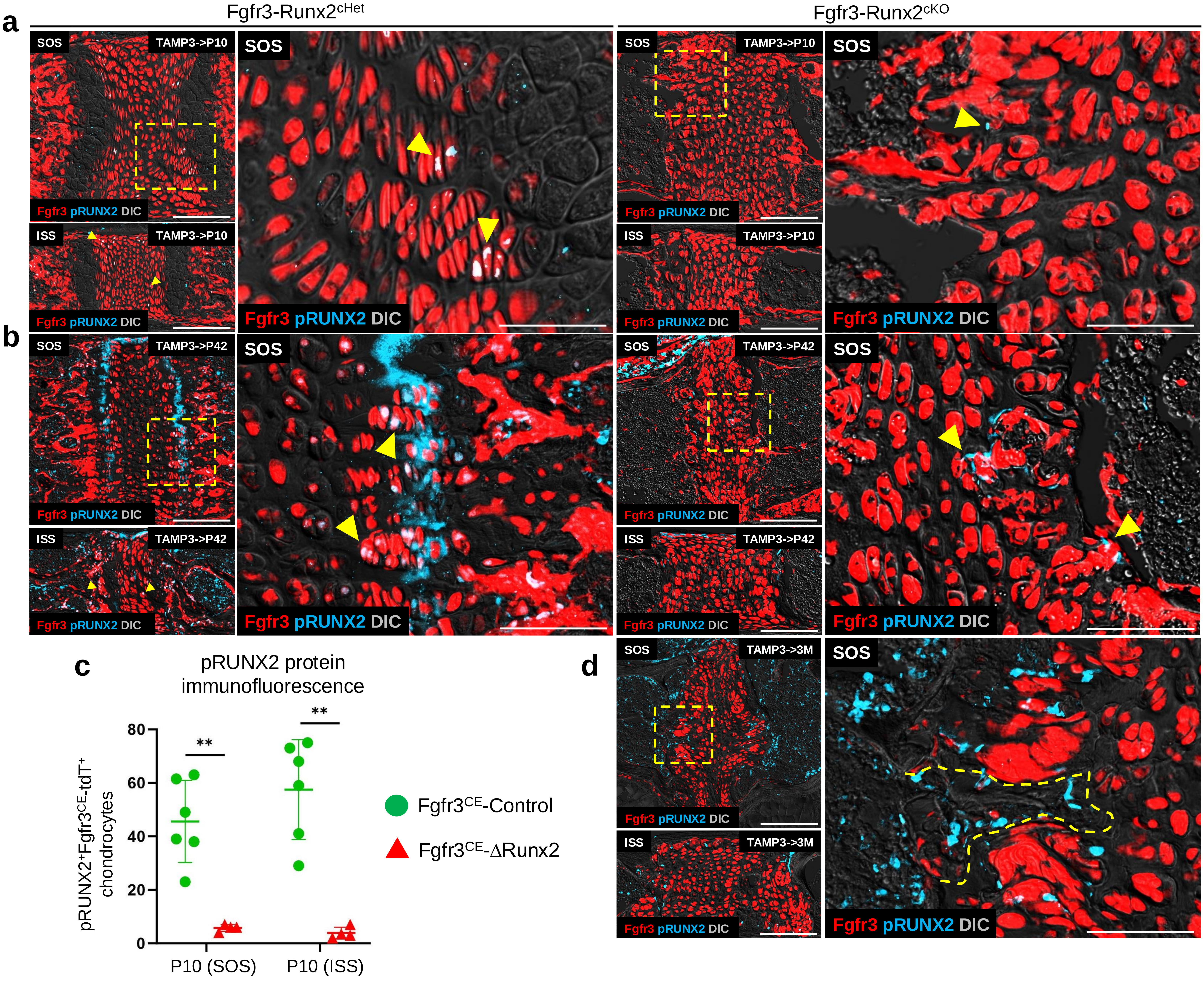

Supplemental Figure 11

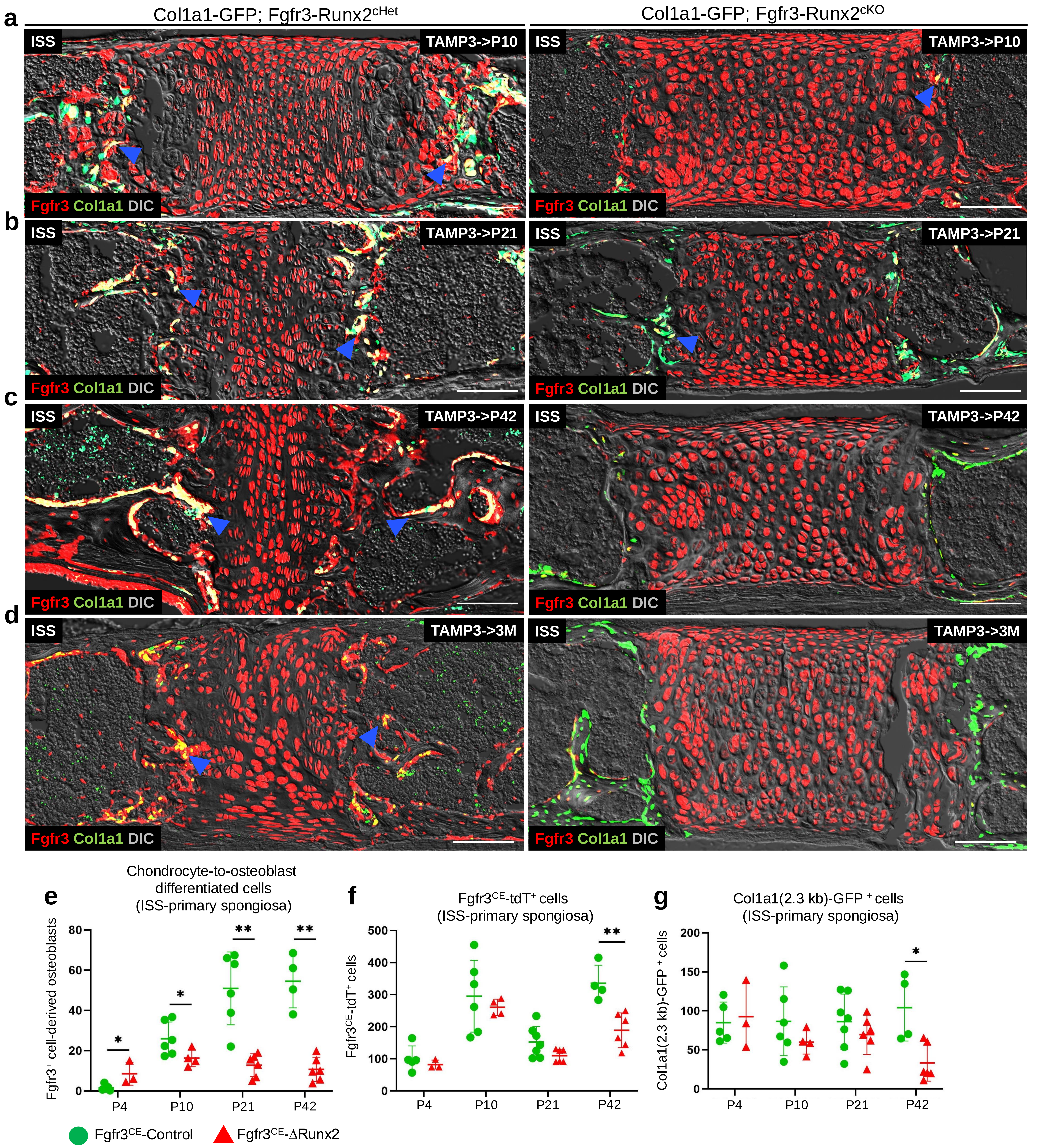

Supplemental Figure 12

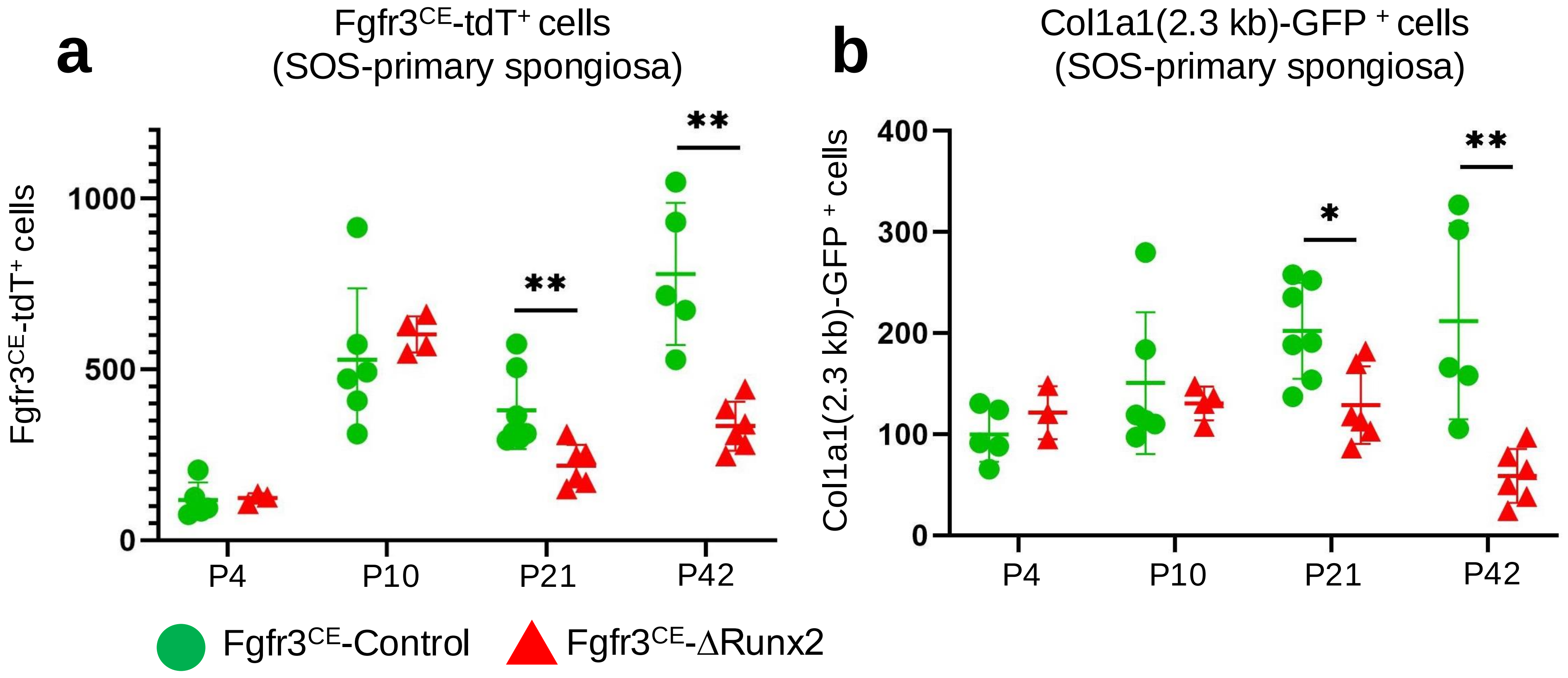

Supplemental Figure 13

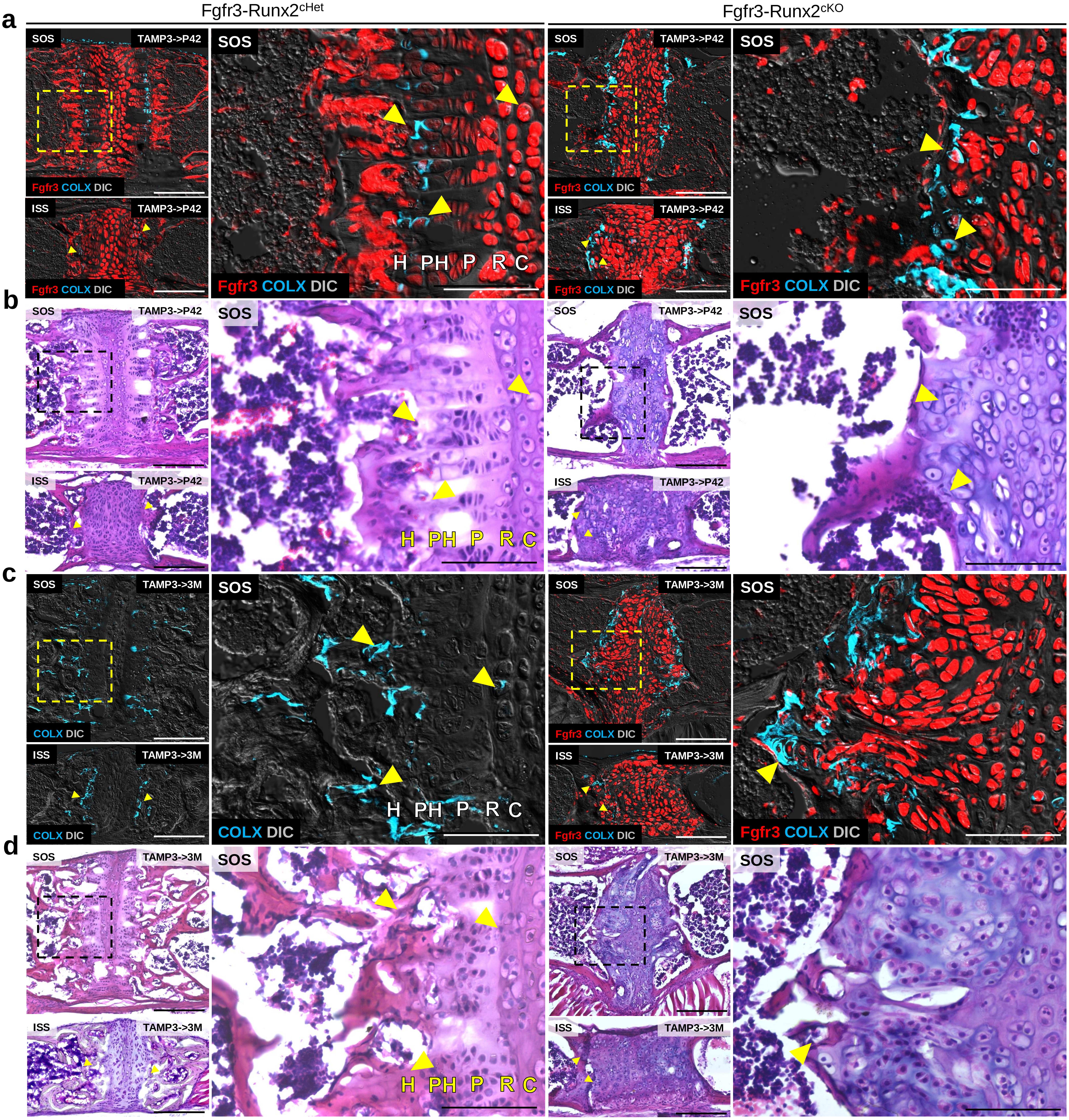

Supplemental Figure 14

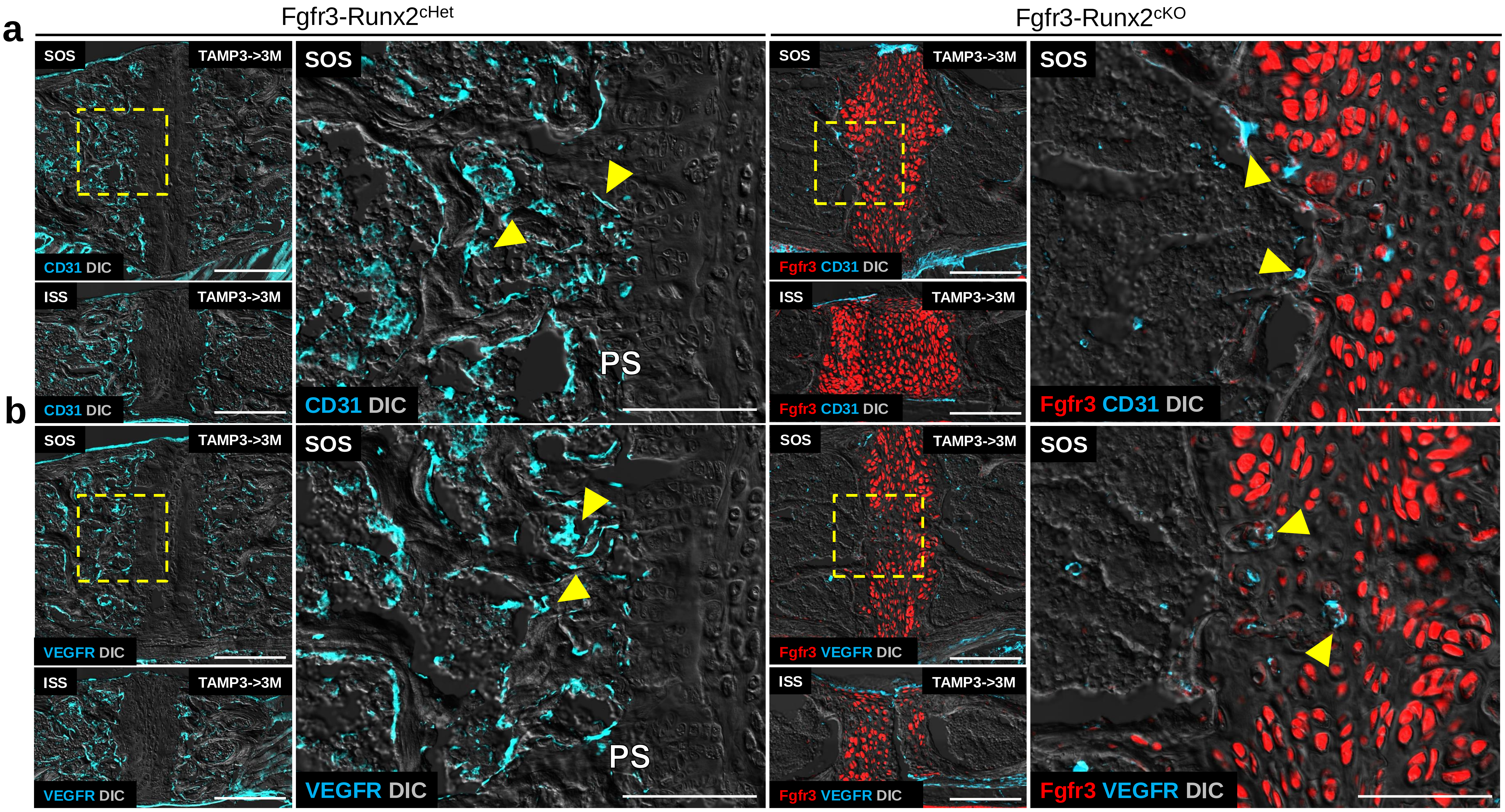

Supplemental Figure 15

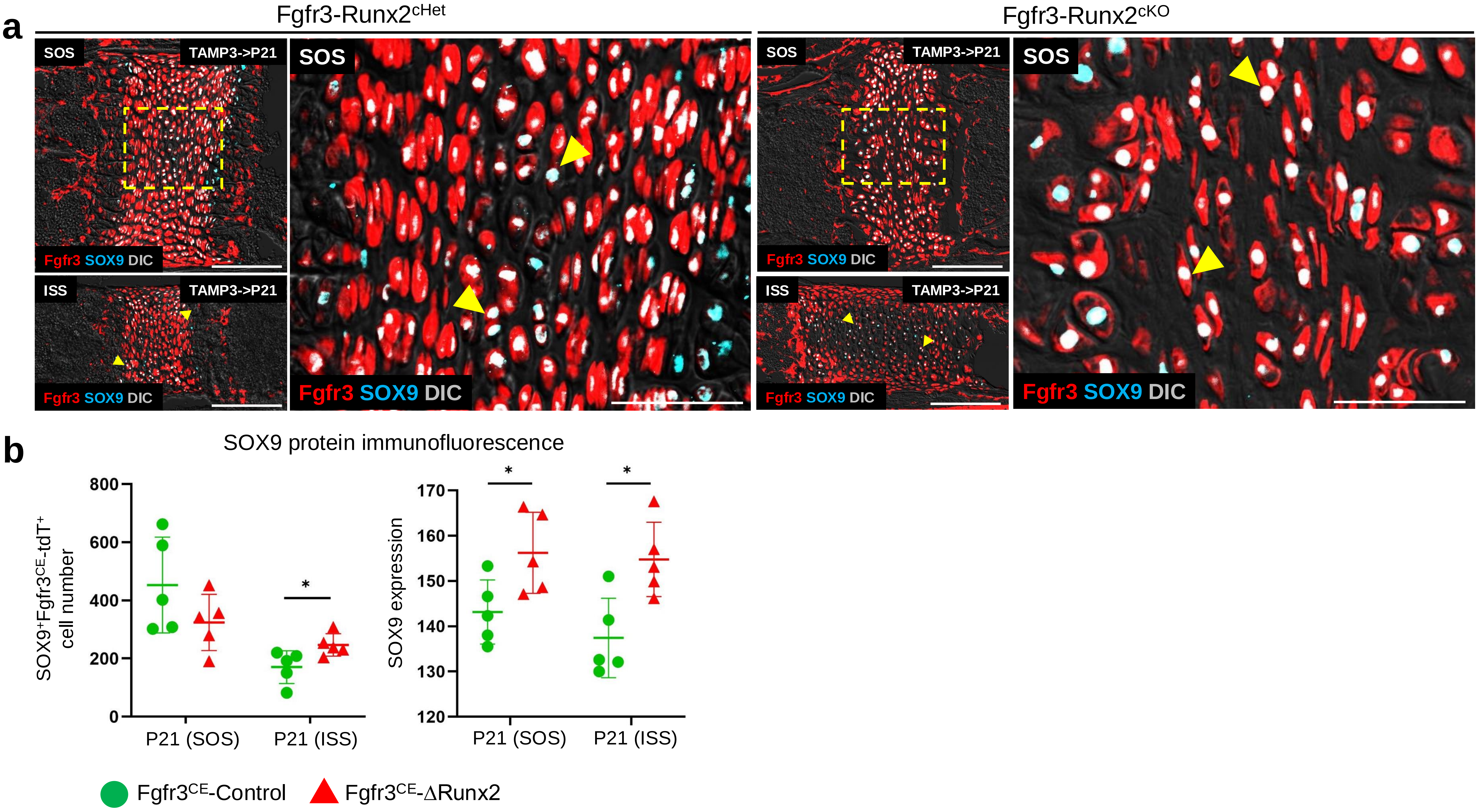

Supplement: Supplementary file 1 — Supplementary Figures [file 41413_2025_426_MOESM1_ESM.pdf]
