# Supplementary material for: RUNX2 is essential for maintaining synchondrosis chondrocytes and cranial base growth
Source: Bone Res. 2025 May 29;13:57. doi: 10.1038/s41413-025-00426-z (PMC12122814; doi:10.1038/s41413-025-00426-z)
Supplement: Supplementary file 2 — Supplementary Figure Legends [file 41413_2025_426_MOESM2_ESM.docx]

**Supplementary Figure Legends**

**Supplemental Figure 1. *Fgfr3-creER*^+^ chondrocytes maintain the long-term architecture of the postnatal SOS.**

**(a, b)** Quantification of Fgfr3^CE^-tdT^+^ **(a)** and Col1a1(2.3 kb)-GFP^+^ **(b)** cells at primary spongiosa at P4 [n=5], P10 [n=6], P21 [n=7], P42 [n=5] collected from serial sections of SOS at all time points. * p<0.05, **p<0.01, Mann-Whitney’s U-test. Data are presented as mean ± s.d.

**(c)** Lineage-tracing of Fgfr3^CE^-tdT^+^ SOS cells following tamoxifen injection at P3 and lineage-traced to 8M. The boxed region is shown in higher magnification. Arrowheads indicate osteoblast-like cells in the central portion of the SOS. Red: Fgfr3^CE^-tdT, gray: DIC. PS: primary spongiosa. Scale bar: 100µm.

**Supplemental Figure 2. *Fgfr3-creER* labels ISS chondrocytes.**

**(a-d)** Lineage-tracing of Col1a1(2.3 kb)-GFP^+^Fgfr3^CE^-tdT^+^ ISS cells following tamoxifen injection at P3 and lineage-traced to P4 (**a**), 10 **(b)**, P21 **(c)**, P42 **(d)** and 3M **(e)**. The boxed region is shown in higher magnification. Blue arrowheads represent Col1a1(2.3 kb)-GFP^+^Fgfr3^CE^-tdT^+^ differentiated cells at primary spongiosa. Red: Fgfr3^CE^-tdT, green: Col1a1(2.3 kb)-GFP, yellow: Col1a1(2.3 kb)-GFP^+^Fgfr3^CE^-tdT^+^, gray: DIC. PS: primary spongiosa. Scale bar: 100µm.

**(f-h)** Quantification of Col1a1(2.3 kb)-GFP^+^Fgfr3^CE^-tdT^+^ **(f)**, Fgfr3^CE^-tdT^+^ **(g)** and Col1a1(2.3 kb)-GFP^+^ **(h)** cells at primary spongiosa at P4 [n=5], P10 [n=6], P21 [n=6 {Col1a1(2.3 kb)-GFP^+^Fgfr3^CE^-tdT^+^} n=7 {Fgfr3^CE^-tdT^+^, Col1a1(2.3 kb)-GFP^+^}], P42 [n=4] collected from serial sections of ISS at all time points. *p<0.05, **p<0.01, Mann-Whitney’s U-test. Data are presented as mean ± s.d.

**Supplemental Figure 3. *Fgfr3-creER* labels AIOS chondrocytes.**

**(a)** Lineage-tracing of Col1a1(2.3 kb)-GFP^+^Fgfr3^CE^-tdT^+^ AIOS cells at P21. The boxed region is shown in higher magnification. Blue arrowheads represent Col1a1(2.3 kb)-GFP^+^Fgfr3^CE^-tdT^+^ differentiated cells at primary spongiosa. Red: Fgfr3^CE^-tdT, green: Col1a1(2.3 kb)-GFP, yellow: Col1a1(2.3 kb)-GFP^+^Fgfr3^CE^-tdT^+^, gray: DIC. PS: primary spongiosa. Scale bar: 100µm.

**Supplemental Figure 4. Craniofacial architecture is disrupted in Fgfr3-Runx2^cKO^** **mice.**

**(a)** Gross morphology of Fgfr3-Runx2^cKO^ **(i)**, Fgfr3-Runx2^cHet^ **(ii)**, *Fgfr3-creER* (control) **(iii)** mice at P42 following tamoxifen injection at P3.

**(b)** Body weight of control, Fgfr3-Runx2^cHet^, Fgfr3-Runx2^cKO^ mice at P42 (males-control [n=4], Fgfr3-Runx2^cHet^ [n=4], Fgfr3-Runx2^cKO^ [n=4]; females-control [n=5], Fgfr3-Runx2^cHet^ [n=6], Fgfr3-Runx2^cKO^ [n=5]) and 3M (males-control [n=5], Fgfr3-Runx2^cHet^ [n=4], Fgfr3-Runx2^cKO^ [n=5]; females-control [n=4], Fgfr3-Runx2^cHet^ [n=5], Fgfr3-Runx2^cKO^ [n=6]).

**(c)** Naso-anal lengths of control and Fgfr3-Runx2^cKO^ mice at P42 (males-control [n=4], Fgfr3-Runx2^cKO^ [n=6]; females-control [n=7], Fgfr3-Runx2^cKO^ [n=6]).

**(d)** Tail lengths of control and Fgfr3-Runx2^cKO^ mice at P42 (males-control [n=4], Fgfr3-Runx2^cKO^ [n=6]; females-control [n=7], Fgfr3-Runx2^cKO^ [n=6]).

**(e)** Representative 2D sagittal view of P42 skull and visualized for quantification. SL: skull length, FL: facial length, CVL: cranial vault length, FH: facial height, CVH: cranial vault height, PS: pre-sphenoid, BS: basi-sphenoid, BO: basi-occipital, SOS: spheno-occipital synchondrosis, ISS: inter-sphenoid synchondrosis.

(**f, g**) Quantification of CVL (**f**) and FL (**g**) at P9 (control [n=6], Fgfr3-Runx2^cHet^ [n=5], Fgfr3-Runx2^cKO^ [n=5]), P42 (males-control [n=4], Fgfr3-Runx2^cHet^ [n=4], Fgfr3-Runx2^cKO^ [n=4]; females-control [n=5], Fgfr3-Runx2^cHet^ [n=6], Fgfr3-Runx2^cKO^ [n=5]) and 3M (males-control [n=5], Fgfr3-Runx2^cHet^ [n=4], Fgfr3-Runx2^cKO^ [n=5]; females-control [n=4], Fgfr3-Runx2^cHet^ [n=5], Fgfr3-Runx2^cKO^ [n=6]).

(**h-m**) Ratios of FL:FH (**h**), CV width (anterior):FH (**i**), FL:CV width (anterior) (**j**), CVL:CVH (**k**), CV width (middle):CVH (**l**) and CVL:CV width (middle) (**m**) at P9 (control [n=6], Fgfr3-Runx2^cHet^ [n=5], Fgfr3-Runx2^cKO^ [n=5]), P42 (males-control [n=4], Fgfr3-Runx2^cHet^ [n=4], Fgfr3-Runx2^cKO^ [n=4]; females-control [n=5], Fgfr3-Runx2^cHet^ [n=6], Fgfr3-Runx2^cKO^ [n=5]) and 3M (males-control [n=5], Fgfr3-Runx2^cHet^ [n=4], Fgfr3-Runx2^cKO^ [n=5]; females-control [n=4], Fgfr3-Runx2^cHet^ [n=5], Fgfr3-Runx2^cKO^ [n=6]). *p<0.05, **p<0.01, Mann-Whitney’s U-test. Data are presented as mean ± s.d.

**Supplemental Figure 5. Skull lengths are shortened, and cranial vault widths are increased in Fgfr3-Runx2^cKO^** **mice.**

**(a, b)** Sagittal (**a**) and dorsal (**b**) 3D-rendered views of control, Fgfr3-Runx2^cHet^, and Fgfr3-Runx2^cKO^ mice at P9 and P42 indicate decreased anteroposterior elongation of the craniofacial complex **(a)** and increased skull length **(b)**, respectively, in Fgfr3-Runx2^cKO^ mice at P42 in males and females. Arrowheads indicate premature fusion of the coronal suture in Fgfr3-Runx2^cKO^ P42 males. Scale bar=1 mm.

**Supplemental Figure 6. Cranial base synchondroses prematurely ossify in Fgfr3-Runx2^cKO^ mice.**

**(a, b)** Dorsal 3D-rendered views of control, Fgfr3-Runx2^cHet^, and Fgfr3-Runx2^cKO^ mice display patency of synchondroses in males and females (**right panels, arrowheads**) at P9 (**a**) that leads to premature ossification of the SOS in P42 Fgfr3-Runx2^cKO^ mice (**b**). Scale bar=1 mm.

**(c-g)** Quantification of PS (**c**), BS (**d**) and BO **(e)** lengths and ISS **(f)** and SOS **(g)** widths at P9 (control [n=6], Fgfr3-Runx2^cHet^ [n=5], Fgfr3-Runx2^cKO^ [n=5]), P42 (males-control [n=4], Fgfr3-Runx2^cHet^ [n=4], Fgfr3-Runx2^cKO^ [n=4]; females-control [n=5], Fgfr3-Runx2^cHet^ [n=6], Fgfr3-Runx2^cKO^ [n=5]) and 3M (males-control [n=5], Fgfr3-Runx2^cHet^ [n=4], Fgfr3-Runx2^cKO^ [n=5]; females-control [n=4], Fgfr3-Runx2^cHet^ [n=5], Fgfr3-Runx2^cKO^ [n=6]). PS: pre-sphenoid, BS: basi-sphenoid, BO: basi-occipital, SOS: spheno-occipital synchondrosis, ISS: inter-sphenoid synchondrosis. *p<0.05, **p<0.01, Mann-Whitney’s U-test. Data are presented as mean ± s.d.

**Supplemental Figure 7. Posterior cranial base is unaffected in Fgfr3-Runx2^cKO^ mice.**

(**a, b**) Dorsal and coronal 3D-rendered views of Fgfr3-Runx2^cHet^, and Fgfr3-Runx2^cKO^ mice at P9 (left), P42 (**middle**) and 3M (**right**) of AIOS and exoccipital bone (**a**), foramen magnum (**b**) and C1-C3 vertebral columns (**b, P9**), respectively, display lack of morphological differences.

(**c**) Representative 2D dorsal view of skull and visualized for quantification. BO: basi-occipital, EXO: exoccipital, AIOS: anterior intraoccipital synchondrosis.

(**d-f**) Quantification of AIOS width (**d**), EXO length (**e**) and FM width (**f**) at P9 (control [n=6], Fgfr3-Runx2^cHet^ [n=5], Fgfr3-Runx2^cKO^ [n=5]), P42 (males-control [n=4], Fgfr3-Runx2^cHet^ [n=4], Fgfr3-Runx2^cKO^ [n=4]; females-control [n=5], Fgfr3-Runx2^cHet^ [n=6], Fgfr3-Runx2^cKO^ [n=5]) and 3M (males-control [n=5], Fgfr3-Runx2^cHet^ [n=4], Fgfr3-Runx2^cKO^ [n=5]; females-control [n=4], Fgfr3-Runx2^cHet^ [n=5], Fgfr3-Runx2^cKO^ [n=6]). *p<0.05, Mann-Whitney’s U-test. Data are presented as mean ± s.d.

**Supplemental Figure 8. Cranial vault width is increased in Fgfr3-Runx2^cKO^ mice.**

**(a)** Coronal μCT scans of calvaria bones at posterior (left panel), middle (center panel), and anterior (right panel) cranial vault positions at 3M in Fgfr3-Runx2^cHet^ and Fgfr3-Runx2^cKO^ mice.

**(b, c)** Quantification of posterior **(b)** and anterior **(c)** cranial vault widths at P9 (control [n=6], Fgfr3-Runx2^cHet^ [n=5], Fgfr3-Runx2^cKO^ [n=5]), P42 (males-control [n=4], Fgfr3-Runx2^cHet^ [n=4], Fgfr3-Runx2^cKO^ [n=4]; females-control [n=5], Fgfr3-Runx2^cHet^ [n=6], Fgfr3-Runx2^cKO^ [n=5]) and 3M (males-control [n=5], Fgfr3-Runx2^cHet^ [n=4], Fgfr3-Runx2^cKO^ [n=5]; females-control [n=4], Fgfr3-Runx2^cHet^ [n=5], Fgfr3-Runx2^cKO^ [n=6]) indicate increases in anterior vault width in Fgfr3-Runx2^cKO^ mice at P42 and 3M in males and females. *p<0.05, **p<0.01, Mann-Whitney’s U-test. Data are presented as mean ± s.d.

**Supplemental Figure 9. *Runx2* inactivation in Fgfr3^+^ cells disrupts cell organization of the AIOS.**

**(a)** Fgfr3-Runx2^cHet^ and Fgfr3-Runx2^cKO^ mice were injected with tamoxifen once at P3 and euthanized at P21 **(c)**. Hematoxylin and eosin staining revealed organized chondrocyte layers in Fgfr3-Runx2^cHet^ mice, showing higher magnification in boxed regions. Fgfr3-Runx2^cKO^ mice displayed disorganization of chondrocyte layers **(right panels)**. Arrowheads indicate short stacks of proliferating columns and resting like cells in the center AIOS **(a, left magnified panel)** and areas of chondrocyte disorganization **(a, right magnified panels)**. R: resting zone, P: proliferating zone, H: hypertrophic zone. Scale bar=100 µm.

**Supplemental Figure 10. pRUNX2 activity is nearly absent in Fgfr3-Runx2^cKO^ synchondroses.**

**(a, b)** Fgfr3-Runx2^cHet^ and Fgfr3-Runx2^cKO^ mice were injected with tamoxifen at P3 and lineage-traced to P10 (a) and P42 (b). Synchondroses were stained with pRUNX2. Boxed regions show higher magnification. pRUNX2^+^Fgfr3^CE^-Control^+^ cells are present in Fgfr3-Runx2^cHet^ proliferating and pre-hypertrophic chondrocytes **(left magnified panels, arrowheads)** but pRUNX2^+^Fgfr3^CE^-∆Runx2^+^ chondrocytes were nearly absent in Fgfr3-Runx2^cKO^ synchondroses **(right magnified panels, arrowheads)**. Red: Fgfr3^CE^-tdT, blue: pRUNX2, gray: DIC. Scale bar: 100 µm.

**(c)** Quantification of pRUNX2^+^Fgfr3^CE^-tdT^+^ synchondrosis chondrocytes at P10 (Fgfr3^CE^-Control n=6, Fgfr3^CE^-∆Runx2 n=4). **p<0.01, Mann-Whitney’s U-test. Data are presented as mean ± s.d.

**(d)** Fgfr3-Runx2^cKO^ mice were injected with tamoxifen at P3 and lineage-traced to 3M. Synchondroses were stained with pRUNX2. Boxed regions show higher magnification. pRUNX2^+^Fgfr3^CE^-tdT^-^ osteoblasts are in the bony defect **(yellow dashed lines)**. Red: Fgfr3^CE^-tdT, blue: pRUNX2, gray: DIC. Scale bar: 100 µm.

**Supplemental Figure 11. *Fgfr3-creER* labels ISS chondrocytes, and RUNX2 promotes their differentiation into osteoblasts.**

**(a-d)** Lineage-tracing of Col1a1(2.3 kb)-GFP^+^Fgfr3^CE^-tdT^+^ ISS cells at P10 **(a)**, P21 **(b)**, P42 **(c)** and 3M **(d)**. Blue arrowheads represent Fgfr3-CE^tdT+^; Col1a1(2.3 kb)-GFP^+^ differentiated cells at primary spongiosa. Red: Fgfr3^CE^-tdT, green: Col1a1(2.3 kb)-GFP, yellow: Col1a1(2.3 kb)-GFP^+^Fgfr3^CE^-tdT^+^, gray: DIC. gray: DIC. Scale bar: 100µm.

**(e-g)** Quantification of Col1a1(2.3 kb)-GFP^+^Fgfr3^CE^-tdT^+^ **(e)**, Fgfr3^CE^-tdT^+^ **(f)** and Col1a1(2.3 kb)-GFP^+^ **(g)** cells at primary spongiosa at P4 (Fgfr3^CE^-Control n=5, Fgfr3^CE^-∆Runx2 n=3), P10 (Fgfr3^CE^-Control n=6, Fgfr3^CE^-∆Runx2 n=4), P21 (Fgfr3^CE^-Control n=6 [Col1a1(2.3 kb)-GFP^+^Fgfr3^CE^-tdT^+^; n=7 [Fgfr3^CE^-tdT^+^, Col1a1(2.3 kb)-GFP^+^], Fgfr3^CE^-∆Runx2 n=6), and P42 (Fgfr3^CE^-Control n=4, Fgfr3^CE^-∆Runx2 n=6) collected from serial sections of ISS at all time points. *p<0.05, **p<0.01, Mann-Whitney’s U-test. Data are presented as mean ± s.d.

**Supplemental Figure 12. Fgfr3-Runx2^cKO^ synchondroses display reduced long-term labeling of Fgfr3^CE^-tdT^+^ chondrocytes.**

**(a, b)** Quantification of Fgfr3^CE^-tdT^+^ **(a)** and Col1a1(2.3 kb)-GFP^+^ **(b)** cells at primary spongiosa at P4 (Fgfr3^CE^-Control n=5, Fgfr3^CE^-∆Runx2 n=3), P10 (Fgfr3^CE^-Control n=6, Fgfr3^CE^-∆Runx2 n=4), P21 (Fgfr3^CE^-Control n=7, Fgfr3^CE^-∆Runx2 n=6) and P42 (Fgfr3^CE^-Control n=5, Fgfr3^CE^-∆Runx2 n=6) collected from serial sections of SOS at all time points. *p<0.05, **p<0.01, Mann-Whitney’s U-test. Data are presented as mean ± s.d.

**Supplemental Figure 13. Fgfr3^CE^-∆Runx2 chondrocytes partially retain hypertrophic characteristic over time.**

(**a, c**) Fgfr3-Runx2^cHet^ and Fgfr3-Runx2^cKO^ mice were injected with a single dose of tamoxifen once at P3 and euthanized at P42 (**a**) and 3M (**c**). Synchondroses were stained with COLX. Boxed regions show higher magnification. COLX^+^Fgfr3^CE^-Control^+^ cells sparsely label Fgfr3-Runx2^cHet^ hypertrophic chondrocytes **(a, c, left magnified panels, arrowheads)**. To a greater extent, COLX^+^Fgfr3^CE^-∆Runx2^+^ chondrocytes robustly label Fgfr3-Runx2^cKO^ synchondroses **(a, b, right magnified panels, arrowheads)**. Red: Fgfr3^CE^-tdT, blue: COLX, gray: DIC. Scale bar: 100 µm.

(**b, d**) Fgfr3-Runx2^cHet^ and Fgfr3-Runx2^cKO^ mice were injected with tamoxifen once at P3 and lineage-traced to P42 **(b)** and 3M **(d)**. Hematoxylin and eosin staining revealed organized chondrocyte layers in Fgfr3-Runx2^cHet^ mice, showing higher magnification in boxed regions. Fgfr3-Runx2^cKO^ mice displayed mineralized areas throughout the SOS's central portion and complete loss of organized chondrocyte layers **(b, d, right panels)**. Arrowheads indicate hypertrophic-like cells in the central and peripheral zones of the SOS **(b, d, left magnified panels)** and areas of chondrocyte disorganization in both Fgfr3-Runx2^cKO^ synchondroses **(b, d, right magnified panels)**. C: central zone, R: resting zone, P: proliferating zone, PH: pre-hypertrophic zone, H: hypertrophic zone. Scale bar=100 µm.

**Supplemental Figure 14. Fgfr3-Runx2^cKO^ synchondroses display reduced angiogenesis.**

(**a, b**) Fgfr3-Runx2^cHet^ and Fgfr3-Runx2^cKO^ mice were injected with a single dose of tamoxifen once at P3 and euthanized at 3M. Synchondroses were stained with CD31 (**a**) and VEGFR (**b**). Boxed regions show higher magnification. Arrowheads indicate robust CD31 and VEGFR expressing endothelial and bone marrow stromal cells and their precursors, respectively, within the marrow compartment in Fgfr3-Runx2^cHet^ synchondroses (**a, b, left magnified panels**). CD31 and VEGFR expression is markedly reduced in Fgfr3-Runx2^cKO^ synchondroses associated with reduced bone formation. Expression tended to localize in Fgfr3^CE^-tdT^-^ areas possibly associated with ‘bony bridge’ formation (**a, b, right magnified panels, arrowheads**). PS: primary spongiosa. Scale bar=100 µm.

**Supplemental Figure 15. SOX9 activity is increased in Fgfr3-Runx2^cKO^ synchondroses.**

**(a)** Fgfr3-Runx2^cHet^ and Fgfr3-Runx2^cKO^ mice were injected with tamoxifen at P3 and lineage-traced to P21. Synchondroses were stained with SOX9 **(a)**. Boxed regions show higher magnification. SOX9^+^Fgfr3^CE^-Control^+^ and SOX9^+^Fgfr3^CE^-∆Runx2^+^ cells are present throughout all chondrocyte layers in both Runx2^cHet^ and Runx2^cKO^ synchondroses **(a, left/right magnified panels, arrowheads)**. Red: Fgfr3^CE^-tdT, blue: SOX9, gray: DIC. Scale bar: 100 µm.

**(b)** Quantification of SOX9^+^Fgfr3^CE^-tdT^+^ synchondrosis chondrocytes **(left graph)** and SOX9^+^ fluorescence intensity **(right graph)** at P21 (Fgfr3^CE^-Control n=5, Fgfr3^CE^-∆Runx2 n=5). *p<0.05, Mann-Whitney’s U-test. Data are presented as mean ± s.d.
